# Novel Resampling Improves Statistical Power for Multiple-Trait QTL Mapping

**DOI:** 10.1534/g3.116.037531

**Published:** 2017-01-06

**Authors:** Riyan Cheng, R. W. Doerge, Justin Borevitz

**Affiliations:** * Research School of Biology, The Australian National University, Acton, Australian Capital Territory 2601, Australia, ARC Center of Excellence in Plant Energy Biology, The Australian National University, Acton, ACT 2601, Australia; † Department of Statistics, Department of Biological Sciences, Carnegie Mellon University, Pittsburgh, PA 15213

**Keywords:** multiple-trait mapping, quantitative trait locus (QTL), resampling, statistical power

## Abstract

Multiple-trait analysis typically employs models that associate a quantitative trait locus (QTL) with all of the traits. As a result, statistical power for QTL detection may not be optimal if the QTL contributes to the phenotypic variation in only a small proportion of the traits. Excluding QTL effects that contribute little to the test statistic can improve statistical power. In this article, we show that an optimal power can be achieved when the number of QTL effects is best estimated, and that a stringent criterion for QTL effect selection may improve power when the number of QTL effects is small but can reduce power otherwise. We investigate strategies for excluding trivial QTL effects, and propose a method that improves statistical power when the number of QTL effects is relatively small, and fairly maintains the power when the number of QTL effects is large. The proposed method first uses resampling techniques to determine the number of nontrivial QTL effects, and then selects QTL effects by the backward elimination procedure for significance test. We also propose a method for testing QTL-trait associations that are desired for biological interpretation in applications. We validate our methods using simulations and *Arabidopsis thaliana* transcript data.

Quantitative trait locus (QTL) mapping has been a powerful tool for dissecting genetic variants underlying quantitative traits in numerous biological studies and breeding programs. There are two primary concerns in QTL mapping. One is the power for QTL identification under a controlled false positive rate, and the other is accuracy of QTL localization. Advanced intercross lines (Darvasi and Soller 1995), collaborative cross (Churchill *et al.* 2004), and heterogeneous stock (Valdar *et al.* 2006) are examples that research efforts have been made to create experimental mapping resources to enhance mapping resolution. In the meantime, various statistical methodologies have been developed to improve statistical power as well as parameter estimation. Joint analysis of multiple complex traits was proposed for QTL mapping in this context as quantitative genetic studies commonly collect data on several to dozens of phenotypes. While multiple traits are usually analyzed separately (referred to as single-trait analysis), there has been plenty of interest in joint analysis of multiple traits over the last two decades. Multivariate analysis of multiple complex traits (referred to as multiple-trait analysis or multitrait analysis) is known for its potential for a higher statistical power and more accurate QTL localization (Korol *et al.* 1995; Jiang and Zeng 1995), and has a wide range of successful applications in various genetic studies such as crops (Wu *et al.* 1999), dairy cattle (Bolormaa *et al.* 2010), model organisms (Zeng *et al.* 2000), diseases (Zhong *et al.* 2010), and other (Lan *et al.* 2006).

The most common methodology for multiple-trait analysis includes multivariate regression (Ronin *et al.* 1995; Knott and Haley 2000), and multiple-trait interval mapping (Jiang and Zeng 1995; Korol *et al.* 1995; 2001). Multiple-trait interval mapping usually relies on multivariate regression models, also when genotypes of a putative QTL are assumed to be known; however, at a putative QTL other than marker loci, the genotypes are only certain with probabilities, and, therefore, the actual model underlying interval mapping is typically a mixture. Generalized linear models, as well as methods that deal with non-normality, also have their applications in multiple-trait analysis (Henshall and Goddard 1999; Lange and Whittaker 2001; Xu *et al.* 2005; Liu *et al.* 2009). In recent years, mixed linear models popular in single-trait analysis have been extended for multiple-trait analysis (Lund *et al.* 2003; Malosetti *et al.* 2008; Hernandez *et al.* 2012; Korte *et al.* 2012). While these multiple-trait approaches generally specify parameters for the effects of a putative QTL on all traits, models that associate different QTL with different traits have been introduced to multiple-trait mapping in both Bayesian and Frequentist frameworks (Verzilli *et al.* 2005; Banerjee *et al.* 2008; Wisser *et al.* 2011; Silva *et al.* 2012).

As indicated above, the common practice of multiple-trait analysis uses multivariate regression models, or models of this type, where parameters are specified for QTL effects on all traits. This corresponds to assuming that a putative QTL is associated with all of the traits, which is usually not true in reality. There are two potential consequences (Brown *et al.* 2014). First, a model with overly excessive QTL effect parameters can reduce the power for QTL detection because the increase in a test statistic due to additional parameters may not compensate for the increase in the degrees of freedom. Second, it is desirable to know which traits are associated with a QTL, and/or which are not; however, multiple-trait analysis that is based on multivariate regression models, or models of this type, does not facilitate such biological interpretation. On the other hand, methods that allow different traits to have different QTL, which facilitates biological interpretation, often focuses on methodological development with little effort to exploit the power for QTL identification (Verzilli *et al.* 2005; Banerjee *et al.* 2008; Wisser *et al.* 2011; Silva *et al.* 2012). Motivated to resolve these limitations, we developed methods for multiple-trait analysis with two aims: to (1) improve statistical power for QTL detection; and (2) derive QTL-trait associations with improved power. We validated our methods using simulations and *Arabidopsis thaliana* transcript data.

## Materials and Methods

Before we proceed to present our methodology, we assume a mapping population of recombinant inbreed lines (RILs) for QTL analysis, and the likelihood ratio test (LRT) statistic for hypothesis testing or model comparison (*i.e.*, we will use LRT statistics in simulations, and real data analysis unless stated otherwise). These assumptions are simply for ease of description of our methodology; however, the methods to develop will not be limited to RILs but will apply to a general mapping population.

We assume a multiple-trait single-QTL model (without covariates)

$$\begin{array}{l} y_{n1}=\beta_{01}+x_{nl}\beta_{l1}+\epsilon_{n1},\\ y_{n2}=\beta_{02}+x_{nl}\beta_{l2}+\epsilon_{n2},\\ \ldots \ldots \\ y_{np}=\beta_{0p}+x_{nl}\beta_{lp}+\epsilon_{np}, \end{array}$$ (M1)

where $y_{nk}$ is the *n*-th (n=1,2,…,N) observation of the *k*-th trait, $\beta_{0k}$ is the intercept, $x_{nl}$ is 1/2, or −1/2 if the genotype of the *l*-th scanning locus (referred to as putative QTL) is AA or aa, $\beta_{lk}$ is the coefficient representing QTL effect, and $\left(\epsilon_{n1},\epsilon_{n2},\ldots ,\epsilon_{np}\right)$ is a *p*-variate random error term. We may assume that $\left(\epsilon_{n1},\epsilon_{n2},\ldots ,\epsilon_{np}\right),$ n=1,2,⋯,N, are independent and follow a normal distribution N(0,Σ) with $\Sigma =\left(\sigma_{ij}\right)$ being a p×p positive definite matrix. In model (M1), we may omit *l* if there is no confusion.

Usually, hypotheses for testing the putative QTL in model (M1) are

$$\begin{array}{l} H_{0}:\beta_{lk}=0\;\text{for}\;\text{all}\;k=1,2,\cdots ,p,\\ H_{1}:\beta_{lk}\neq 0\;\text{for}\;\text{any}\;k=1,2,\cdots ,p. \end{array}$$ (H1)

If we assume the *k*-th trait, $k\in \bar{K}\subset \left\{1,2,\cdots ,p\right\},$ is not associated with the putative QTL, then model (M1) becomes

$$\begin{array}{l} y_{nk}=\beta_{0k}+\epsilon_{nk}\;\text{if}\;k\in \bar{K},\text{or}\\ y_{nk}=\beta_{0k}+x_{nl}\beta_{lk}+\epsilon_{nk}\;\text{if}\;k\notin \bar{K} \end{array}$$ (M2)

and the hypotheses for testing the putative QTL are

$$\begin{array}{l} H_{0}:\beta_{lk}=0\;\text{for}\;\text{all}\;k=1,2,\cdots ,p,\\ H_{1}:\beta_{lk}=0\;\text{for}\;\text{all}\;k\in \bar{K},\text{and}\;\beta_{lk}\neq 0\;\text{for}\;\text{any}\;k\in \left\{1,2,\cdots ,p\right\}\backslash \bar{K} \end{array}$$ (H2)

if we use model (M1), or

$$\begin{array}{l} H_{0}:\beta_{lk}=0\;\text{for}\;\text{all}\;k\in \left\{1,2,\cdots ,p\right\}\backslash \bar{K},\\ H_{1}:\beta_{lk}\neq 0\;\text{for}\;\text{any}\;k\in \left\{1,2,\cdots ,p\right\}\backslash \bar{K} \end{array}$$ (H2a)

if we look at model (M2).

We next present a few methods that can be used to detect QTL: maximum bootstrap power (MBP), test QTL effects individually (Indv), remove trivial QTL effects sequentially (Seq), and model selection by Bayesian information criterion (BIC) (BIC*_δ_*). While we propose MBP for multiple-trait analysis, we consider the others for the sake of comparison, as they are natural and intuitive choices when we are interested in QTL-trait associations in applications. Especially, we compare them with the most common approach, *i.e.*, test hypotheses (H1) for QTL, which we denote by “All”. These methods, except Indv, do not necessarily adequately provide information about QTL-trait associations, but instead can yield excessive false positive QTL-trait associations even though the type I error rate of QTL is appropriately controlled at a nominal significance level. Therefore, we also propose a method that tests QTL-trait associations after a scanning locus is identified as QTL. At the end of this section, we describe a real data set and simulation settings.

### MBP

It is known that multiple-trait analysis is likely, but not always, more powerful than single-trait analysis, depending on QTL effects and the residual correlation structure of the traits (Jiang and Zeng 1995; Korol *et al.* 1995). In Cheng *et al.* (2013), we argued that addition of a trait to multiple-trait analysis can lead to a reduced statistical power for QTL identification if, given other traits, it contributes little to the test statistic. Therefore, we proposed to select a subset of traits for multiple-trait analysis as follows: (1) determine the maximum number, *K* (up to the total number of traits), of traits to be selected; (2) take 1000 (say) nonparametric bootstrap samples, and estimate the (pseudo) statistical power and its SE for k=1,2,⋯,K “best” traits, denoted by $p_{k}$ and $e_{k}$ respectively; (3) choose the largest $k^{*}\left(\leq K\right)$ such that $p_{k*}\geq p_{k*+1}-e_{k*+1}$ and $p_{k*-1}<p_{k*}-e_{k*}.$ The key point is to select a number of traits such that any additional trait contributes little to the statistical power according to certain criterion. This method improves statistical power as we showed in Cheng *et al.* (2013). On the other hand, we showed that addition of a trait increases statistical power for QTL detection if this trait is reasonably correlated with traits of interest, and is not associated with the QTL. As a useful application, we can include additional traits that satisfy the above conditions when we do not have sufficient power to detect QTL underlying traits of our interest. In such a case, the QTL is not associated with all traits in the model.

Now, we consider a situation where we are interested in joint analysis of multiple traits without excluding any of them or including additional traits. In practice, a QTL may be only associated with some, but not all, of the traits, *i.e.*, some $\beta_{lk}$ values in model (M1) are zero. As shown in the *Results* section, power for QTL detection is not optimal if the model contains parameters representing QTL effects on all of the traits, but not all of the traits are influenced by the QTL. Then, we may improve power by excluding “trivial” QTL effects that are either actually zero or statistically nonsignificant. How to exclude trivial QTL effects is our focus in this paper. We adopt the idea for variable selection of traits in Cheng *et al.* (2013) as introduced above, and propose a procedure as follows.

1. Obtain significance threshold $\lambda_{k}\left(\alpha \right)$ for the best *k* (k=1,2,…,p) effects at genome-wide significance level, *α*, where *p* is the total number of traits;
2. Take *B* (say 250) nonparametric bootstrap samples, and, for the *b*-th bootstrap sample, obtain the test statistic, $T_{k}^{\left(b\right)},$ for the best *k* (k=1,2,…,p) effects at a putative QTL;
3. For each *k*, calculate the proportion, $f_{k}$, of the bootstrap samples where $T_{k}^{\left(b\right)}>\lambda_{k}\left(\alpha \right),$ b=1,2,⋯,B, *i.e.*, $f_{k}=\sum_{b=1}^{B}I_{T_{k}^{\left(b\right)}>\lambda_{k}\left(\alpha \right)}/B$ where $I_{T_{k}^{\left(b\right)}>\lambda_{k}\left(\alpha \right)}=1$ if $T_{k}^{\left(b\right)}>\lambda_{k}\left(\alpha \right)$, or 0 otherwise;
4. Find $k_{0}$ such that $k_{0}=\arg\max_{1\leq k\leq p}f_{k}$ if $k_{0}$ is unique, or take the integer part of the average of the smallest and the largest $k_{0}$value otherwise;
5. Claim a QTL if $T_{k_{0}}>\lambda_{k_{0}}\left(\alpha \right),$ where $T_{k}$ (k=1,2,⋯,p) is the test statistic for the best *k* effects at the putative QTL in a genome scan.

Note that $T_{k},$ $T_{k}^{\left(b\right)}$, and $f_{k}$ are specific to a scanning locus, *e.g.*, $T_{lk}$ will be used instead of $T_{k}$ if the best *k* effects at the *l*-th of *L* scanning loci are tested for QTL. Obviously, $P\left(T_{lk}<\lambda_{k}\left(\alpha \right),l=1,2,\cdots ,L\right)\geq 1-\alpha$ for any k=1,2,⋯,p under that null hypothesis of no QTL as $\lambda_{k}\left(\alpha \right)$ is the genome-wide threshold at significance level *α* for the best *k* QTL effects, and, therefore, if $k_{0}$ is chosen purely by chance, we also have $P\left(T_{lk_{0}}<\lambda_{k_{0}}\left(\alpha \right),l=1,2,\cdots ,L\right)\geq 1-\alpha .$ The best effects of a putative QTL are the remaining effects after trivial ones are excluded, say, by forward selection, backward elimination, or stepwise selection. Backward elimination is often preferable as it usually has a better performance than forward selection in our current situation, and is less computationally intensive than stepwise backward selection. In (3), we can use a more stringent criterion than $\lambda_{k}\left(\alpha \right)$ because $f_{k}$ can easily reach 1 when QTL effects are moderately large, so that it is difficult to choose a desirable $k_{0}$ in (4). For example, we can replace $\lambda_{k}\left(\alpha \right)$ with $\lambda_{k}\left(\beta \right)$ such that $P\left(T_{lk}<\lambda_{k}\left(\beta \right),l=1,2,\cdots ,L,k=1,2,\cdots ,p\right)\geq 1-\alpha$ under the null hypothesis of no QTL. The threshold $\lambda_{k}\left(\beta \right)$values correct multiplicity in both *l* and *k* by adjusting the significance level from *α* to *β* (generally α>β), which can be accomplished numerically. We can further assume $P\left(T_{l1}<\lambda_{1}\left(\beta \right),l=1,2,\ldots ,L\right)=P\left(T_{l2}<\lambda_{2}\left(\beta \right),l=1,2,\ldots ,L\right)=\ldots =P\left(T_{lp}<\lambda_{p}\left(\beta \right),l=1,2,\ldots ,L\right)$ if we do not give preference to any k=1,2,…,p. If $k_{0}$ is not unique in (4), which is often the case when $f_{k}$values reach 1, the smallest $k_{0}$ can result in underestimation of the number of nontrivial effects, whereas the largest can lead to overestimation. Therefore, we take a trade-off to guard against a serious bias.

Steps 2–4 in the above procedure determine nontrivial effects, and step 5 assumes trivial effects are zero and tests for QTL using model (M2). We note that bootstrap samples generally produce a greater $f_{k}$ than independent samples, that is, bootstrap $f_{k}$ overestimates the type I error rate if there is no QTL, or the power if a QTL exists (see Supplemental Material, File S1, for more information). If we still regard $f_{k}$ an estimate of power at a putative QTL, then the above procedure seeks $k_{0}$ that maximizes (pseudo) statistical power at the putative QTL, which we refer to as MBP.

### Indv

The MBP method excludes trivial QTL effects using bootstrap samples. The number of nontrivial QTL effects, $k_{0},$ is determined by an ensemble of bootstrap samples rather than a single sample. Alternatively, we may be tempted to exclude trivial QTL effects individually if the corresponding test statistics are smaller than a cutoff, *τ*. Specifically, we consider the test statistics, $T_{lk}$, in model (M1) with hypotheses

$$H_{0}:\beta_{lk}=0\;\mathrm{vs}\text{.}\;H_{1}:\beta_{lk}\neq 0$$ (H3)

k=1,2,⋯,p, and regard $\beta_{lk}$ as trivial if $T_{lk}<\tau .$ This subsequently determines $k_{0}$, and testing for QTL existence is then based on the best $k_{0}$ effects. Though simple yet intuitive, this unfortunately does not work well. A large cutoff *τ* can result in a great loss of power, especially if the number of QTL effects is relatively large, whereas a small *τ* may lead to inflated type I error rates, or little gain in power.

If we aim to identify associations between a putative QTL and traits of interest, we can directly test whether QTL effects on the traits are zero, which in turn indicates QTL existence. It is intuitively reasonable to test multiple QTL effects one at a time in a multiple-trait framework. There are two options. One is to test an effect given all other effects in the model, and the other is to test an effect with all other effects being excluded from the model. The former is somewhat similar to looking at the type III sums of squares or the *z*-test statistics constructed from estimates and their SE in multiple regression, which are usually reported in statistic software applications. The latter is similar to single-trait analysis, but is now under a multiple-trait framework. As seen in the *Results* section, this will lead to a damage in power due to underfitting if the number of QTL effects is >1. Therefore, this is not a good option, and will not be taken for further consideration. The first option, denoted Indv, will be referred to when we test QTL effects individually. The hypotheses are those in (H3). To control the genome-wide type I error rate at significance level *α*, the threshold τ(α) should be such that $P\left(\max_{l=1,2,\ldots ,L,\;k=1,2,\ldots ,p}T_{lk}>\tau \left(\alpha \right)\right]=P\left[T_{lk}>\tau \left(\alpha \right),l=1,2,\ldots ,L,\;k=1,2,\ldots ,p\right)\leq \alpha$ where $T_{lk}$ is assumed to be the LRT statistic for testing the effect, $\beta_{lk}$ in model (M1), of a putative QTL at the *l*-th scanning locus on the *k*-th trait, given the effects of the putative QTL on all other traits, that is, τ(α) is adjusted for multiplicity in both traits and scanning loci.

### Seq

Again, assume we are interested in QTL-trait associations, which, in turn, indicates QTL existence. In statistics, it is not rare to test multiple effects one after another until a significant effect is disclosed. However, selection bias can be induced if the process sequentially searches for, and removes, the least significant effect among all the remaining effects. We may choose to ignore, or take into account, selection bias when we determine significance thresholds. In case selection bias is ignored, a simple way is to use a single threshold with Bonferroni correction, which is basically the BIC*_δ_* method we present next, and is stringent for the least significant QTL effects. We will not consider this option, but instead can refer to the BIC*_δ_* method for its performance in terms of power. To take selection bias into account, we can estimate thresholds through the permutation test, where the permuted data are analyzed in the same way as the original data (*i.e.*, the data that we permute). The significance threshold can be adjusted for multiplicity in the same way for MBP and Indv. We denote this method by Seq.

### BIC*_δ_*

Model selection is a useful tool in multiple-QTL mapping, in either a Frequentist (Kao *et al.* 1999; Broman and Speed 2002; Arends *et al.* 2010; Wang *et al.* 2011) or a Bayesian (Yi *et al.* 2003) framework. While modern model selection techniques such as shrinkage methodology have appealing applications in genetic analysis (Whittaker *et al.* 2000; Xu 2003; Wang *et al.* 2005), the traditional backward elimination and stepwise selection approaches are still favored for their simplicity and interpretability. Broman and Speed (2002) recommends backward elimination along with BIC*_δ_* for multiple-QTL mapping. BIC*_δ_* is different from the well-known BIC only in the penalty, and Broman and Speed (2002) suggest a penalty that is associated with the genome-wide threshold rather than the sample size. In our current situation of multiple-trait analysis, we consider BIC*_δ_*, and obtain a penalty as follows if we are interested in QTL-trait associations.

1. Permute the genotype data;
2. Select the best effect for the permuted data by one-step forward selection starting from the model without QTL effects;
3. Repeat 1) and 2) for 1000 (say) times, and take the (1−α) quantile, τ(α), of the test statistics adjusted for multiplicity in scanning loci for the best effect.

The hypotheses in (2) are

$$\begin{array}{l} H_{0}:\beta_{lt}=0\;\text{for}\;\text{all}\;t=1,2,\cdots ,p,\\ H_{1}:\beta_{lk}\neq 0,\text{and}\;\beta_{lt}=0\;\text{for}\;\text{all}\;t\in \left\{1,2,\cdots ,p\right\}\backslash \left\{k\right\} \end{array}$$ (H4)

k=1,2,⋯,p. The threshold τ(α) in (3) is such that $P\left(\max_{l=1,2,\ldots ,L,k=1,2,\ldots ,p}T_{lk}>\tau \left(\alpha \right)\right)=P\left(T_{lk}>\tau \left(\alpha \right),\;l=1,2,\ldots ,L,\;k=1,2,\ldots ,p\right)\leq \alpha$, where $T_{lk}$ is the statistic that tests hypotheses (H4) in the permuted data. This penalty is expected to control the type I error rate at the genome-wide significance level, *α*, if forward selection is performed for QTL identification, and, as seen in the *Results* section, works reasonably well if backward elimination is implemented.

### Test individual QTL effects after a QTL is identified

The MBP is expected to reasonably control the type I error rate for QTL identification at a nominal significance level; however, it does not necessarily control the type I error rate for a QTL effect at the nominal significance level. As a matter of fact, $k_{0}$ tends to overestimate the number of QTL effects. Overfitting is not necessarily a bad thing. First, MBP aims to remove some trivial effects to improve power, rather than estimate the number of nonzero QTL effects. Second, as shown in the *Results* section, overestimation of the number of QTL effects does not damage power as much as underestimation, though it may not lead to an optimal power. To test individual QTL effects at a controlled type I error rate, *α*, we can proceed with the following testing procedure (denoted idv).

1. Calculate the test statistic, say $\chi^{2},$ for a QTL effect given all other effects;
2. Claim the QTL effect is not zero if $\chi^{2}>\chi_{v}^{2}\left(1-\alpha /k_{0}\right)$, where *υ* is the degrees of freedom and $k_{0}$ is the number of nontrivial QTL effects.

Here, we assume LRT for ease of description, and its asymptotic $\chi^{2}$-distribution. We also assume the trivial effects, which are determined by MBP, are not statistically significantly different from zero, and thus excluded from testing in (2). Denote the trivial set by $\bar{K}.$ The hypotheses are

$$\begin{array}{l} H_{0}:\beta_{lt}=0\;\text{for}\;\text{all}\;t=1,2,\cdots ,p,\\ H_{1}:\beta_{lk}\neq 0,\text{and}\;\beta_{lt}=0\;\text{for}\;\text{all}\;t\in \bar{K} \end{array}$$ (H5)

$k\in \left\{1,2,\cdots ,p\right\}\backslash \bar{K}.$ The multiplicity in *k* is corrected by the Bonferroni procedure in (2). As a matter of fact, inclusion of trivial effects does not appreciably promote power, but potentially contributes false positives. To sum up, we can combine MBP and idv to test QTL-trait associations as follows.

1. Test whether a locus is a QTL by the MBP procedure;
2. If the locus is a QTL, go with the above procedure, idv, to test whether a QTL effect is zero.

Denote this two step-procedure by MBP+idv. Since MBP+idv is based on MBP, identified individual QTL effects will not result in a greater genome-wide type I error rate for QTL detection. Similarly, we can combine any of the other methods with idv to test individual QTL effects, as demonstrated in the *Results* section.

### Resampling

Unlike in simulations, where we can generate independent data sets, we typically have only one set of data in an application. While we can make inference just from one set of real data, modern statistical methodology, such as bagging, often takes advantage of an ensemble, *e.g.*, (nonparametric) bootstrap samples, to improve accuracy and reduce uncertainty. In this regard, Valdar *et al.* (2009) proposes model averaging for QTL mapping. Resampling techniques have a wide range of applications, *e.g.*, assessment of sample statistics, such as variance, hypothesis testing, and model validation. The most common resampling methods include permutation (Fisher 1935), bootstrapping (Efron 1979), and subsampling. Subsampling draws a subset of the data without replacement. Jackknife is such a resampling method, and, like bootstrap, is usually used to assess variance and bias of an estimate. While the conventional jackknife takes delete-1 samples, Shao and Wu (1989) propose delete-*d* jackknife for sample statistics, such as median, for which the delete-1 jackknife estimate is not consistent.

In MBP, we use resampling, bootstrapping, to determine the number of nontrivial QTL effects. We now consider resampling for comparing methods, in terms of both power and making inference in real data applications. Suppose we have *B* subsamples, and, for the *b*-th subsample, $S^{\left(b\right)}=\max_{1\leq l\leq L}S_{l}^{\left(b\right)}$, where $S_{l}^{\left(b\right)}$ is the test statistic at the *l*-th scanning locus, and ζ(α) is the threshold at genome-wide significance level *α*. Define $\hat{p}=\sum_{1\leq b\leq B}I_{S^{\left(b\right)}>\zeta \left(\alpha \right)}/B$ where $I_{S^{\left(b\right)}>\zeta \left(\alpha \right)}=1$ if $S^{\left(b\right)}>\zeta \left(\alpha \right)$ or 0 otherwise. The statistic $\hat{p}$ can be used to test whether there is QTL. If the subsamples are replaced by independent replicate samples, $\hat{p}$ estimates the type I error rate or statistical power, and asymptotically follows a normal distribution if *B* is reasonably large. However, subsamples are not independent, and $\hat{p}$ is not asymptotically normal (see File S1). The distribution of $\hat{p}$ can be estimated by using simulations. The subsamples may be jackknife samples, whereas bootstrap does not seem to be a good choice in our current situation (see File S1, Table S1, and Figure S5). For convenience, we call $\hat{p}$ pseudo type I error rate or pseudo power, or simply relative frequency. We note that $\hat{p}$ is positively related to *p*-values; however, we cannot estimate *p*-values sufficiently accurately by a limited number of simulations to disclose the advantage of a method over another in terms of power. The above $\hat{p}$ can also be defined at scanning locus *l* as ${\hat{p}}_{l}=\sum_{1\leq b\leq B}I_{S_{l}^{\left(b\right)}>\zeta \left(\alpha \right)}/B.$ Depending on the context, $\hat{p}$ or ${\hat{p}}_{l}$ may be relevant to, say, the best $k_{0}$ QTL effects.

### Gene transcript data and simulation settings

Simulation studies are often used to check an idea or validate a method in scientific research, and may be essential if there is a lack of theory. Proper settings are crucially important in simulation studies. While it is easy to set up simulations if there are only two traits, it will be hard to come up with a suitable variance–covariance structure of traits when the number of the traits is relatively large. Therefore, we considered the expression trait (e-trait) data that we analyzed in Cheng *et al.* (2013). Briefly, 211 RILs were derived from two parental inbred *A. thaliana* accessions, Bayreuth-0 (Bay-0) and Shahdara (Sha), by selfing (Loudet *et al.* 2002; West *et al.* 2007), and their gene transcripts (“E-TABM-126” in the ArrayExpress database) were generated by Affymetrix microarray technology (Kliebenstein *et al.* 2006). We chose the transcripts of 16 genes from a pathway as phenotypic data. The genotypic data consisted of 95 markers (West *et al.* 2006) across five chromosomes, with the maximum genetic distance between two adjacent markers being 10.944 cM, the minimum 2.224 cM, and the median 4.771 cM. This data are suited for demonstration of multiple-trait methodology. The sample correlations between the e-traits range from 0.02 to 0.96, with a median 0.66. In addition, the known locations of the genes allow us to roughly verify the results of real data analysis.

The data were analyzed in Cheng *et al.* (2013) using both single-trait and multiple-trait approaches, and several QTL were identified. Single-trait analysis indicated that QTL at markers 3, 10, 37, and 78 were possibly associated with one trait, whereas QTL at markers 27 and 89 seemed to influence multiple traits. In this study, we chose one marker from each of the five chromosomes, and used their genotypic data directly (coded as 0.5 or −0.5 if the genotype was AA or aa, respectively). These five selected markers were 3, 27, 46, 65, and 89. (Markers 46 and 65 were located near the middle of chromosomes 3 and 4, respectively.) In simulations, phenotypic data were generated as follows. We fitted a multivariate multiple regression model with the responses being the 16 e-traits, and predictors being the five chosen marker variables. Denote the estimated intercepts ${\hat{\beta }}_{0}=\left({\hat{\beta }}_{01},{\hat{\beta }}_{02},\ldots ,{\hat{\beta }}_{0,16}\right),$ the estimated effects ${\hat{\beta }}_{l}=\left({\hat{\beta }}_{l1},{\hat{\beta }}_{l2},\ldots ,{\hat{\beta }}_{l,16}\right)$, corresponding to marker *l* (l=3,27,46,65,89), and the estimated residual variance-covariance $\hat{\Sigma }={\left({\hat{\sigma }}_{ij}\right)}_{16\times 16}.$ Let $\gamma_{lk}={\hat{\beta }}_{lk}/3$ if $\left|{\hat{\beta }}_{lk}\right|/\sqrt{{\hat{\beta }}_{kk}}\geq 0.45$, or 0 otherwise. The resulting $\gamma_{l}=\left(\gamma_{l1},\gamma_{l2},\ldots ,\gamma_{l,16}\right)$ had 1, 11, 0, 3, or 7 nonzero elements for l=3, 27, 46, 65, or 89. We redefined $\gamma_{46,k}={\hat{\beta }}_{27,k}/3$ (*i.e.*, there was no truncation). Then, $\gamma_{46}$ had 16 nonzero elements. We then generated the *i*-th phenotypic observation $y_{i}={\hat{\beta }}_{0}+\epsilon_{i}$ when we looked at type I error rates or $y_{i}={\hat{\beta }}_{0}+\sum_{l\in \left\{3,27,46,65,89\right\}}x_{ij}\gamma_{l}+\epsilon_{i}$ when we compared methods for statistical power, where $\epsilon_{i}\overset{i.i.d}{∼}N\left(0,\hat{\sum }\right)$ and $x_{ij}$ was the coded genotype of the *i*-th individual at marker *l*. In the power study, the variation in a simulated trait explained by a QTL ranged from 0.20 to 5.9% (excluding zero effects; Figure S1).

Note that we truncated $\hat{\beta }$ values in order to have different numbers of nonzero effects (NZE), and scaled them so that the estimated power would not be too large (*e.g.*, nearly 100%) or too small (*e.g.*, nearly 0%), to disguise the differences among the methods that we compared. There is no room (or need) for improvement on power that is nearly 100%, whereas, if power is too low, even an increase of 15% (say) in power may not be disclosed, or may prove to be significant, by a limited number of simulations. Moreover, it is not common in QTL analysis of quantitative traits that QTL effects are so large that they can be identified in nearly all similar experiments. As it was not easy to simulate reasonable QTL effects and correlation structure, we took advantage of the variance–covariance structure in the data, and used the e-traits as a starting point for generating phenotypic data in our simulation studies.

### Data availability

As stated above, the gene transcript data can be obtained from the ArrayExpress database (query ID “E-TABM-126”) and the genetic marker data was published in West *et al.* 2006. File S1 in Cheng *et al.* (2013) provides information about the 16 genes, and a genetic map of the 95 markers. Both genetic marker data and phenotypic data are also accessible from the webpage of the Borevitz laboratory at the Australian National University https://borevitzlab.anu.edu.au/resources/association-studies/.

## Results

### The number of QTL effects and statistical power

**Figure 1:**
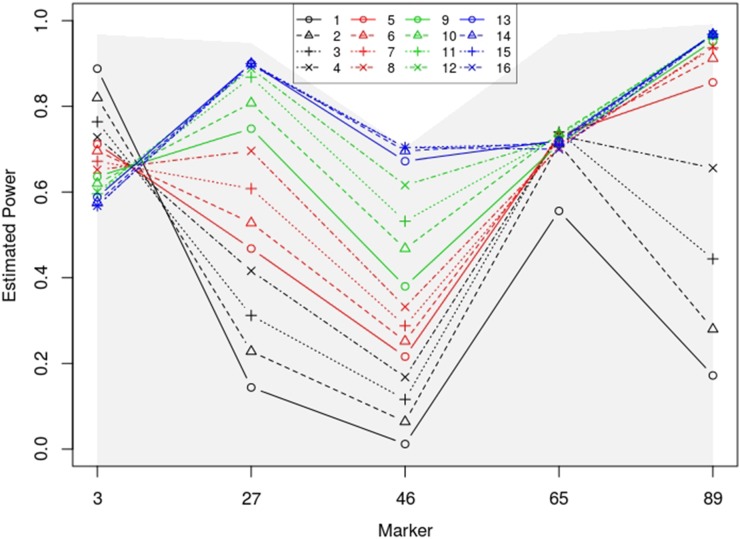
Estimated statistical power for identifying five simulated QTL at genome-wide significance level α=0.05 using various numbers of the best QTL effects, or exactly all the NZE (shaded area). The numbers of nonzero QTL effects at markers 3, 27, 46, 65, and 89 are 1, 11, 16, 3, and 7, respectively.

A QTL may or may not influence all traits of interest. However, the most common practice of multiple-trait mapping does not make this distinction. For instance, the multivariate simple regression model, which is often used in multiple-trait analysis, has parameters representing QTL effects on all of the response components. The power for QTL detection may not be optimal if only a small portion of the traits are influenced by the QTL. Figure 1 demonstrates this point (refer to simulation settings for power study) by showing the power for various numbers of the “best” QTL effects, which are selected by the backward elimination procedure, and for only NZE to be fitted in the model to identify a putative QTL. NZE is the most powerful, but, in real applications, it is unfortunately impossible to know exactly if a scanning locus is a QTL, or which traits are associated with a QTL. In all the other cases where model selection is involved, an optimal power is achieved when the number of the best QTL effects is near the number of NZE. The power decreases at marker 3, where the QTL has an effect only on one trait, as the number of the best QTL effects increases. At the other markers, the gain in power is negligible or null (markers 27 and 89), or somewhat negative (marker 65) after the number of selected best effects reaches the number of true NZEs. We note that underfitting QTL effects generally damages the power for QTL detection, while overfitting may result in a lower power. As in other statistical applications, power is a primary concern in QTL mapping studies. It is therefore worth exploring methodology for removing trivial QTL effects to potentially gain power for QTL identification.

### Simulation results

We conducted 250 simulations to estimate type I error rates or statistical power. In each simulation, significance thresholds were estimated via the permutation test (Churchill and Doerge 1994), with 1200 permutations of the genotypic data, and 250 bootstrap samples were taken to determine the number of the best (*i.e.*, nontrivial) QTL effects. The genome-wide significance level was 0.05 when we compared different methods in terms of type I error rates and statistical power. We expected the estimated type I error rates to be comparable, and fall by ∼0.05 for all methods, so as to not only avoid report excessive false positives but also assure a fair comparison of different methods. When we looked at the power of a method, we primarily compared with the case where all the effects of a putative QTL were included in the model and tested together (denoted by All), unless we clearly state otherwise. We used the LRT statistics as stated in the *Materials and Methods* section.

**Figure 2:**
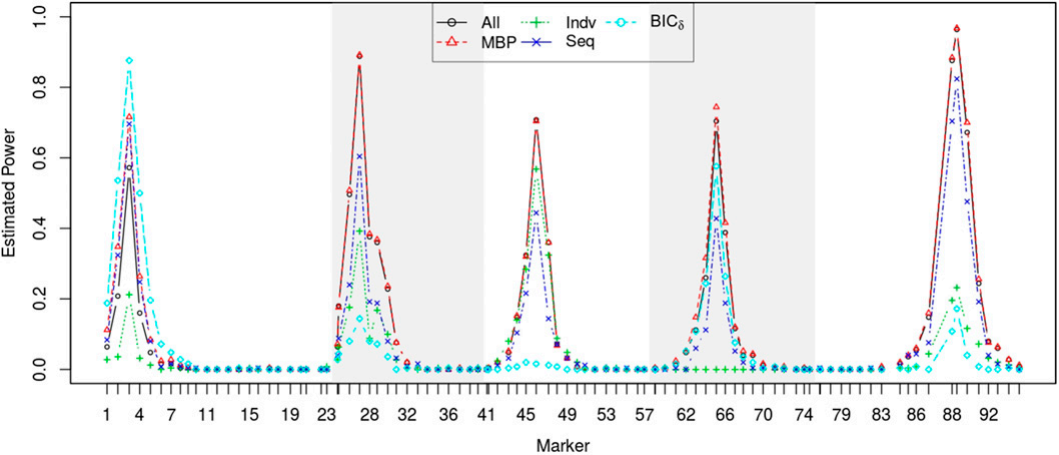
Relative frequency over 250 simulations that a marker was identified as a QTL by five methods: All, MBP, Indv, Seq, and BIC*_δ_* at genome-wide significance level 0.05. Five chromosomes are indicated by light-gray colored shading.

Table 1 displays the estimated genome-wide type I error rates. As expected, all of them were reasonably around the nominal significance level 0.05. Figure 2 shows the estimated statistical power at each scanning locus at genome-wide significance level 0.05. Estimated power was basically the same for both MBP and All at markers that were relatively far away from any of markers 3, 27, 46, 65, and 89 where QTL were simulated, and that were near marker 46 where QTL had effects on all of the 16 traits. Otherwise, MBP tended to be more powerful than All. The estimated power for MBP was larger by 14.4% at marker 3, where only one QTL effect was simulated, by up to 5.6% near marker 65, where three QTL effects were simulated, and by 2.8% near marker 89, where seven QTL effects were simulated.

**Table 1:** Genome-wide type I error rate and its SE estimated from 250 simulations for five methods All, MBP, Indv, Seq, and BIC*_δ_*

All	MBP	Indv	Seq	BIC*_δ_*
0.048 (0.014)	0.056 (0.015)	0.036 (0.012)	0.04 (0.012)	0.06 (0.015)

We now compare Indv, Seq, and BIC*_δ_* with All for QTL detection, though the comparison is more relevant to QTL-trait associations. Indv was less powerful than All, especially at markers 3, 27, 65, and 89. While we might blame overfitting at marker 3, or underfitting at markers 27 and 89, it had little power to identify QTL at marker 65, whose contribution to heritability was small (see Figure 2). Interestingly, its power at marker 46, where none of the QTL effects were zero, was still lower than that of a joint test of all effects. This indicates that all NZEs are best tested together. Seq was more powerful than All at marker 3 but less powerful in other regions. This may be partially related to the problem of underfitting or overfitting. Actually, there are many ways to determine thresholds for Seq such that each can control the genome-wide type I error rate, but lead to a different result that reflects a trade-off between underfitting and overfitting in different situations. BIC*_δ_* was the most powerful at marker 3, where there was only one QTL effect, but was the least powerful when the number of QTL effects was intermediate or large (and lots of QTL effects were relatively small), as BIC*_δ_* can cause serious underfitting.

**Figure 3:**
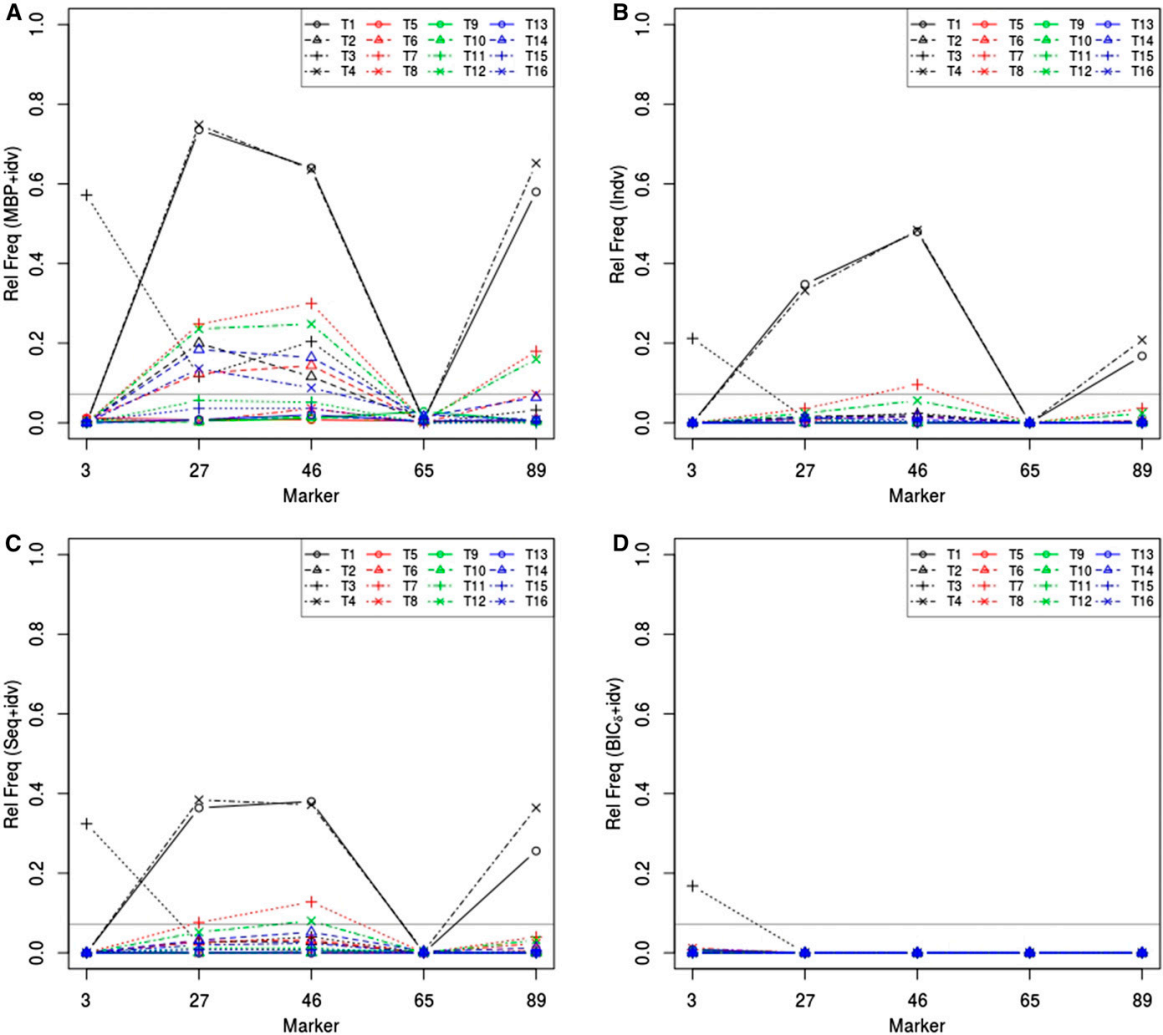
Relative frequency over 250 simulations that a scanning locus was identified as a QTL for each trait by using (A) MBP+idv, (B) Indv, (C) Seq+idv, and (D) BIC*_δ_*+idv. The horizontal dotted line represents $0.05+1.645\sqrt{0.05\times \left(1-0.05\right)/250}\approx 0.0727.$

Indv, Seq, and BIC*_δ_* tested scanning loci for QTL by imposing a penalty, or cutoff, on the contributions of individual QTL effects to the test statistic. However, Seq and BIC*_δ_* could not appropriately tell if a QTL was associated with a trait (see Figure S2). Actually, Seq yielded excessive false positives of QTL-trait associations. To derive QTL-trait associations, we need to proceed with further hypothesis testing, *e.g.*, use the previously proposed method, idv after All, MBP, Seq, or BIC*_δ_*. Indv does not need further testing to provide adequate information about QTL-trait associations because the control of the genome-wide type I error rate for QTL identification is based on identification of individuals QTL-trait associations, and the multiplicity in QTL effects is already accounted for. Figure 3 displays the relative frequency that marker 3, 27, 46, 65, or 89 was identified as a QTL underlying individual traits by using MBP+idv, Indv, Seq+idv, or BIC*_δ_*+idv. There was no longer any concern about excessive false positives. The advantage of MBP+idv over Indv, Seq+idv, or BIC*_δ_*+idv was striking in terms of power.

**Figure 4:**
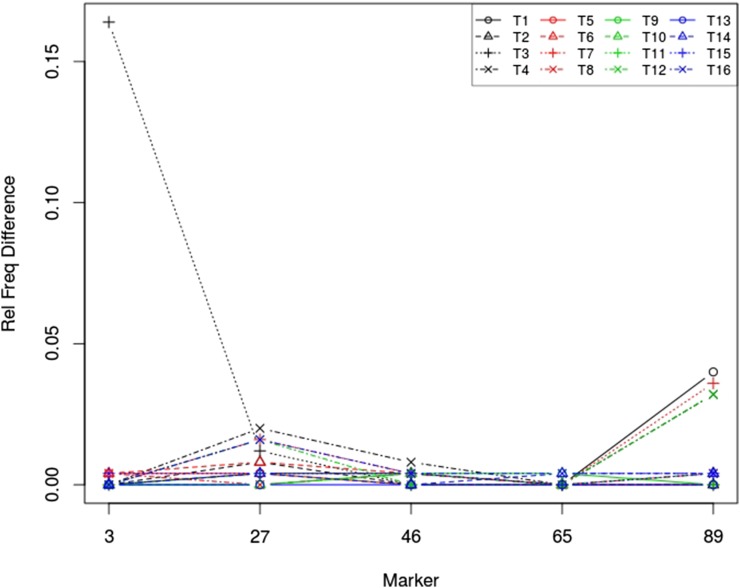
Difference in the relative frequencies over 250 simulations that a scanning locus was identified as a QTL for traits T1, T2, …, T16, by using MBP+idv and All+idv.

Figure 4 displays the difference in the relative frequencies that a scanning locus was identified as a QTL for individual traits by using MBP+idv and All+idv. The gain by using MBP over All was apparent at markers 3, 27, and 89. There were 16 QTL effects at marker 46, so it was not of much help to remove trivial effects. QTL at marker 65 explained little variation in the traits, and, consequently, the difference was negligible.

In the above simulation study, we preselected five markers and estimated their effects ${\hat{\beta }}_{l}=\left({\hat{\beta }}_{l1},{\hat{\beta }}_{l2},\ldots ,{\hat{\beta }}_{j,16}\right),$ l=3,27,46,65,89, and then adjusted ${\hat{\beta }}_{l}$ values to generate QTL. We performed additional simulations to further validate our methods. The settings were the same as above except (1) the scale was 1/4; (2) the scale was 1/2; or (3) the scale was still 1/3 but the sign of ${\hat{\beta }}_{lk}$ was randomly assigned. Results supported the above conclusions, *e.g.*, there were no excessive false positives of QTL-trait associations even when simulated QTL were identified over nearly 100% of the simulations (Figure S6, B2). We further simulated two traits with various configurations for QTL effects and correlation structures. Results again supported our proposed method (see details in File S1, Tables S2, S3, S4 and S5 and Figures S6, S7 and S8).

### Real data example

We applied the above methods to the e-trait data. Genome-wide significance thresholds were estimated via the permutation test with 1200 permutations of the genotypic data. Each of the 211 jackknife samples of the e-trait data, for which we took 250 bootstrap samples to derive the number of the best QTL effects, was analyzed by using All, BMP, Indv, Seq, and BIC*_δ_* and the relative frequency—proportion of the jackknife samples that a scanning locus was identified as a QTL—was obtained for each of the methods (Figure S3). The relative frequency reached 1 in a significant portion of the genome due to large QTL effects. This disguised the advantage of one method over another. However, the benefit of using BMP over All was apparent when we looked at individual QTL-trait associations: the relative frequency that a scanning locus was identified by MBP+idv as a QTL underling a trait was greater (by up to 55.9%) in many regions (Figure S4). The relative frequency obtained from jackknife samples is not the statistical power, but can indicate whether a method is more powerful than another.

**Figure 5:**
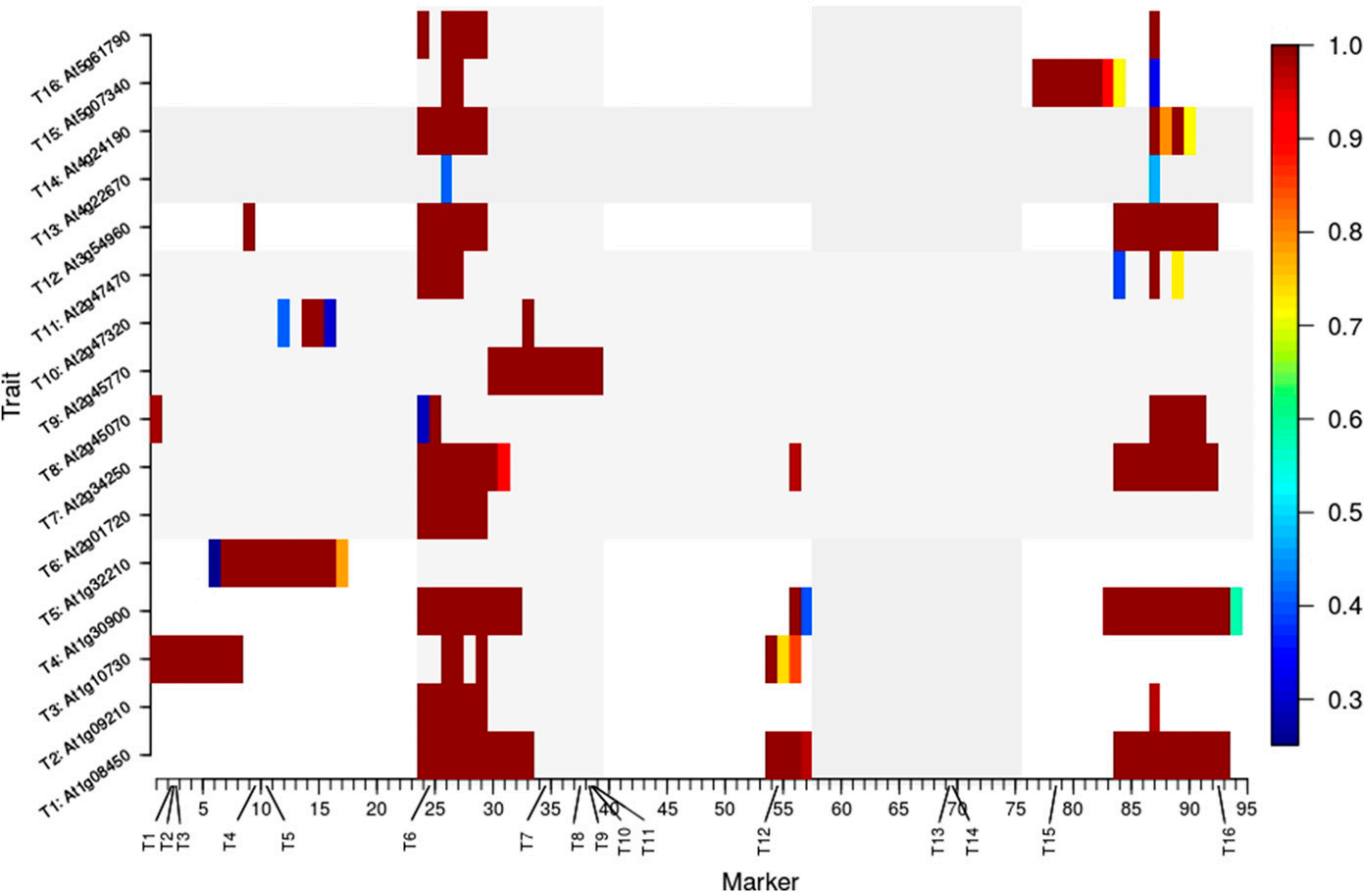
Relative frequency over 211 jackknife samples of the e-trait data that a scanning locus was identified by MBP+idv as a QTL for individual traits at genome-wide significance level 0.05. Relative frequency was zeroed if it was <0.25. The 16 genes are given as the labels on the vertical axis, and their approximate locations are indicated on the horizontal axis.

Figure 5 displays the relative frequency, zeroed if <0.25 (which should control the type I error rate of QTL under 0.05; see File S1 for more information), over 211 jackknife samples of the e-trait data that a scanning locus was identified by MBP+idv as a QTL for individual traits at genome-wide significance level 0.05. This provides useful information for the study of *cis*-acting and *trans*-acting elements, and potentially identify new genes in addition to the existing network of the 16 genes, about which we will not go into detail as it is not the focus of this article.

## Discussion

Multiple-trait analysis has long been advocated for QTL mapping, but its application is somewhat limited due to several possible reasons. First, multiple-trait analysis may not be as computationally easy as single-trait analysis, *e.g.*, when mixed-effect models are fitted. Second, a main motivation for multiple-trait analysis is a potential gain in power for QTL detection, *i.e.*, multiple-trait analysis potentially has a higher statistical power than single-trait analysis by taking advantage of the residual correlation structure among the traits. Unfortunately, multiple-trait analysis is not always more powerful but depends on QTL effects, and the correlation structure among the traits (Korol *et al.* 1995; Jiang and Zeng 1995; Wu *et al.* 1999). Inclusion of a trait in multiple-trait analysis may not be justified in terms of power for QTL detection, as the presence of other traits can greatly reduce its unique information about the QTL. To overcome this limitation, Cheng *et al.* (2013) proposed a variable selection approach, *i.e.*, to choose a subset of traits for multiple-trait analysis in such a way that any trait in the subset is not redundant given the others, and demonstrated that the proposed method achieves a higher power. Third, multiple-trait analysis in applications usually relies on multivariate regression models, or models of this type, and does not readily provide information on QTL-trait associations to facilitate biological interpretation.

Statistical power is a primary concern with respect to hypothesis testing in statistical applications. This work exploits the potential gain in statistical power from multiple-trait analysis. Complementary to Cheng *et al.* (2013), it deals with how to select QTL effects given a number of traits of interest all being included in multiple-trait analysis. The MBP method we proposed first determines the number of nontrivial effects of a putative QTL, and then fits the best QTL effects in the model such that additional effects will contribute little to, or even have an adverse impact on, QTL detection. The gain of MBP in power tends to increase as the proportion of trivial effects of a putative QTL increases. The proposed method is expected to be generally applicable. On one hand, it is not typical that a QTL will influence all traits of interest. On the other hand, we may intentionally add traits that are closely correlated with a trait of interest, but is not associated with a QTL underlying the trait of interest, to increase power for detecting the QTL (Cheng *et al.* 2013). In such cases, a statistical model that associates a QTL with all traits is not parsimonious, and, consequently, has a suboptimal power to identify the QTL. As another aim of this work, we addressed how to derive QTL-trait associations, as it is desirable to know which traits are influenced by a QTL, and which are not. We proposed a two-step procedure, MBP+idv, for this purpose. First, we test by MBP if a scanning locus is a QTL. Second, we test which traits are associated with a QTL. The second step uses information about the number of nontrivial QTL effects from the first step, such that it can adjust to get less stringent significance thresholds for multiple testing. As a result, MBP+idv is able to further improve statistical power for testing QTL-trait associations, in addition to the gain from MBP. We validated our methods by theoretical reasoning, simulations, and real data.

MBP searches a model space that contains the full model for All (*i.e.*, all QTL effect parameters are included in the model), and judges a QTL effect based on its contribution to the test statistic for All. Therefore, all that matters is how much a QTL effect contributes given other QTL effects. The performance of MBP relies on how well trivial QTL effects are excluded. A hard cut-off leads to either overfitting or underfitting, and thus does not work well for a general situation where QTL effects and the number of QTL effects can be either relatively large or small. In contrast, an ensemble of bootstrap samples provides adaptive selection criteria. Nonetheless, MBP overestimates QTL effects. This guards against underfitting, which can greatly hurt power but may not ultimately improve power due to overfitting. How to best exclude trivial QTL effects is an open question. As a shortcoming, MBP is computationally intensive, which can be a problem if the number of scanning loci is large. It is desirable to develop a method that is less computationally intensive.

We also explored a few other intuitive methods: Indv, Seq, and BIC*_δ_*. These can be a natural choice when we are interested in QTL-trait associations in applications. There are many ways to choose thresholds and penalties for Seq and BIC*_δ_*. For instance, penalties can be adapted to the number of the best QTL effects in the model by using corresponding significance thresholds rather than a fixed penalty in BIC*_δ_*. This will improve power if the number of nontrivial QTL effects is relatively large, but, as a trade-off, reduce power otherwise. We note that inference about QTL-trait associations based on Seq and BIC*_δ_* can be unsatisfactory (Figure S2, C and D). As mentioned previously, underfitting of QTL effects can be more problematic than overfitting, especially when model selection is involved. BIC*_δ_* with a fixed penalty generally leads to underfitting since small QTL effects can be excluded easily by the stringent penalty. This, however, is not always in line with our expectation. Our simulation study provides an example that a scanning locus contributes little or no variation to a trait can be more frequently identified as a QTL for the trait (Figure S2D). An explanation for such insensible results would be that the test statistic depends not only on QTL effects and correlation structure, but also on what NZEs should be excluded from the model, which leads to a certain amount of bias.

Lastly, we described our method using quantitative traits, RILs, and linear models, but the principles and methodology should apply to qualitative traits, other mapping populations, and other models, such as linear mixed-effect models, which are now popular in genome-wide association studies.

The analysis was performed by using *R* package “qtlmt,” which is intended for multiple-trait multiple-QTL analysis (currently suitable for backcross and RIL populations) and is available on *R* CRAN (https://cran.r-project.org/web/packages/qtlmt/index.html). *R* code for this study is available from the webpage of the Borevitz laboratory at the Australian National University.

## Supplementary Material

Supplemental material is available online at www.g3journal.org/lookup/suppl/doi:10.1534/g3.116.037531/-/DC1.
