# Supplementary figures and images for: Novel Resampling Improves Statistical Power for Multiple-Trait QTL Mapping
Source: G3 (Bethesda). 2017 Jan 6;7(3):813–22. doi: 10.1534/g3.116.037531 (PMC5345711; doi:10.1534/g3.116.037531)

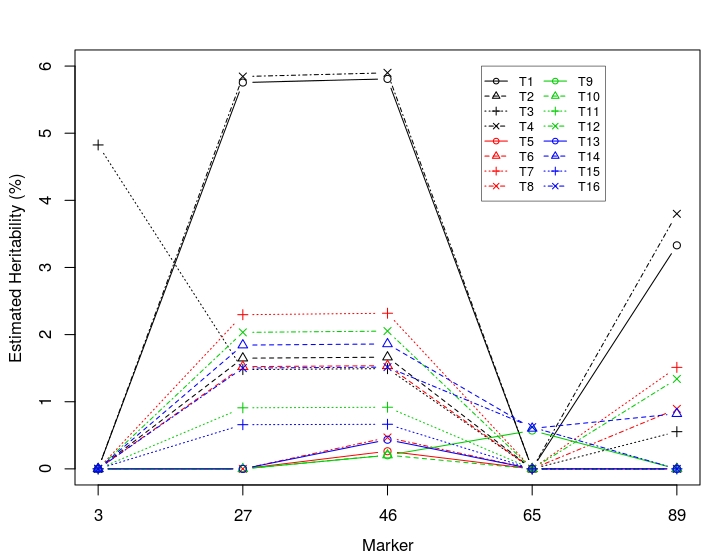

Supplement: Supplementary file 1 [file 813FigureS1.jpg]

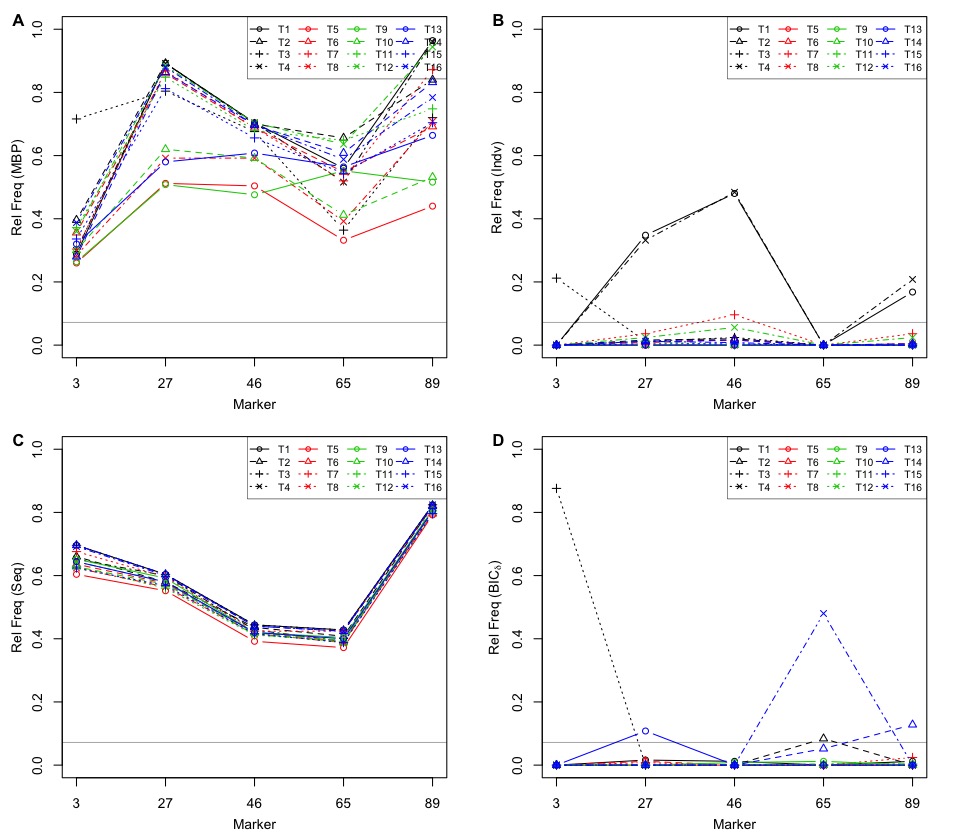

Supplement: Supplementary file 2 [file 813FigureS2.jpg]

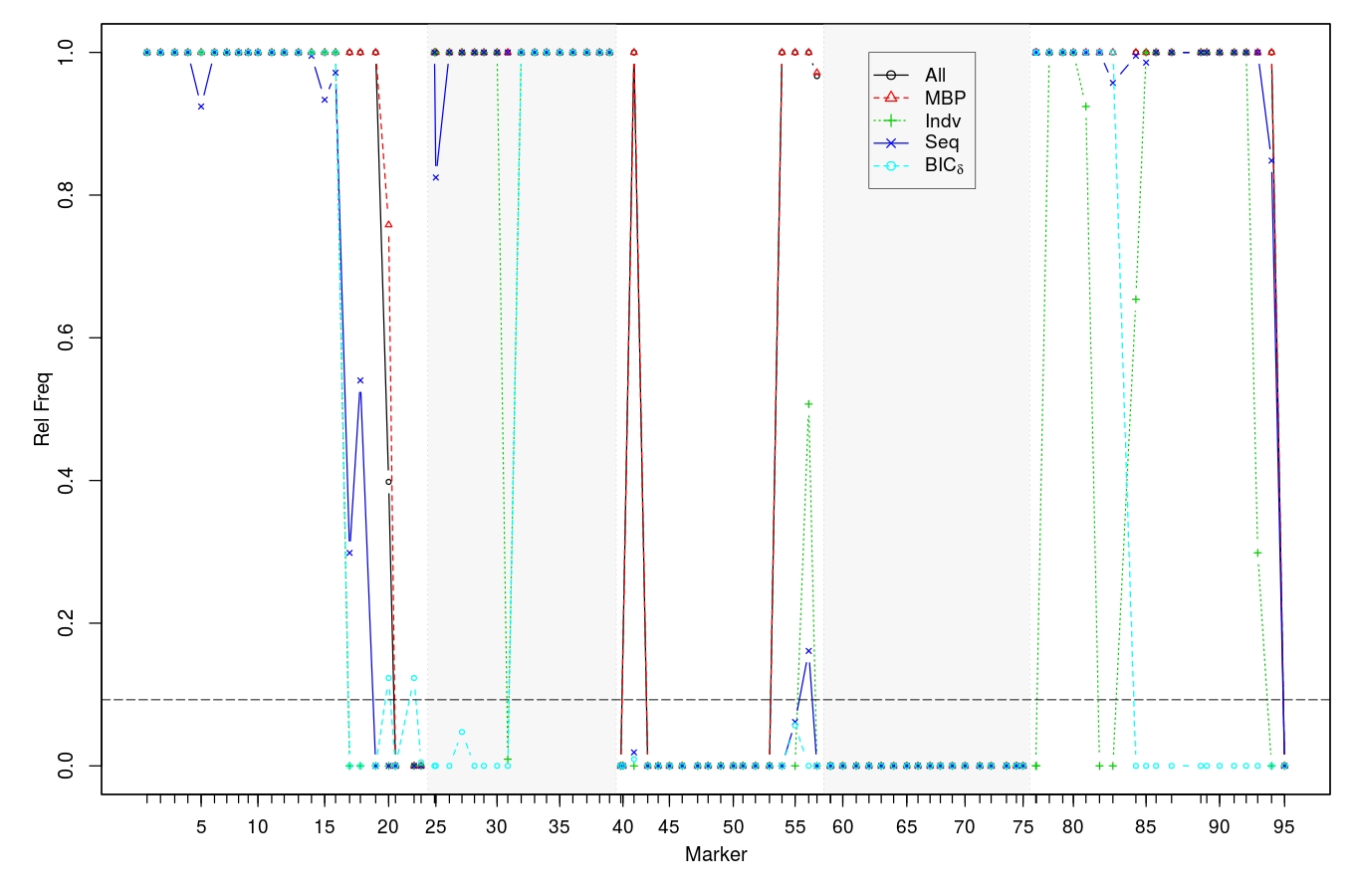

Supplement: Supplementary file 3 [file 813FigureS3.jpg]

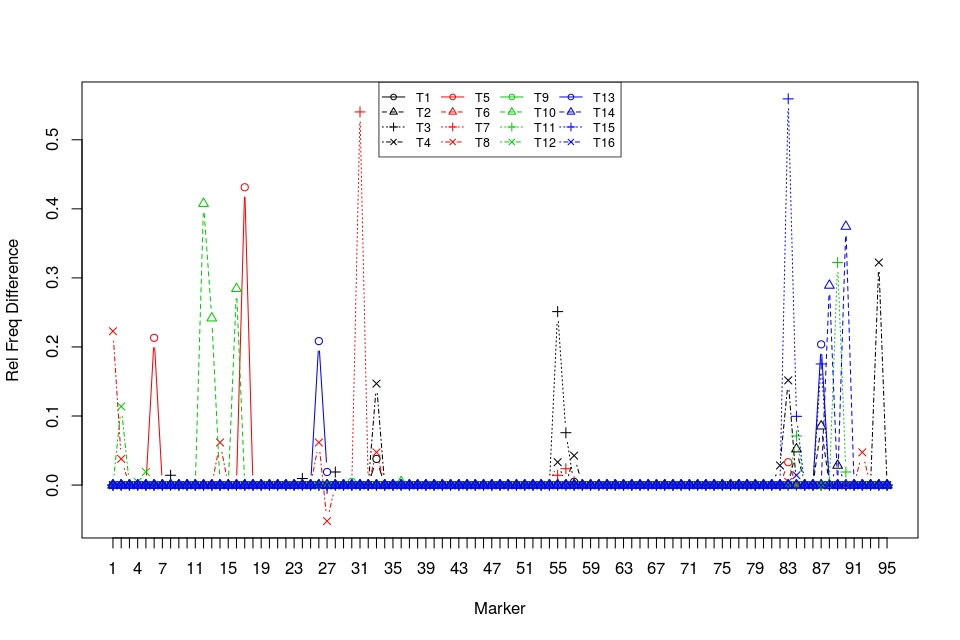

Supplement: Supplementary file 4 [file 813FigureS4.jpg]

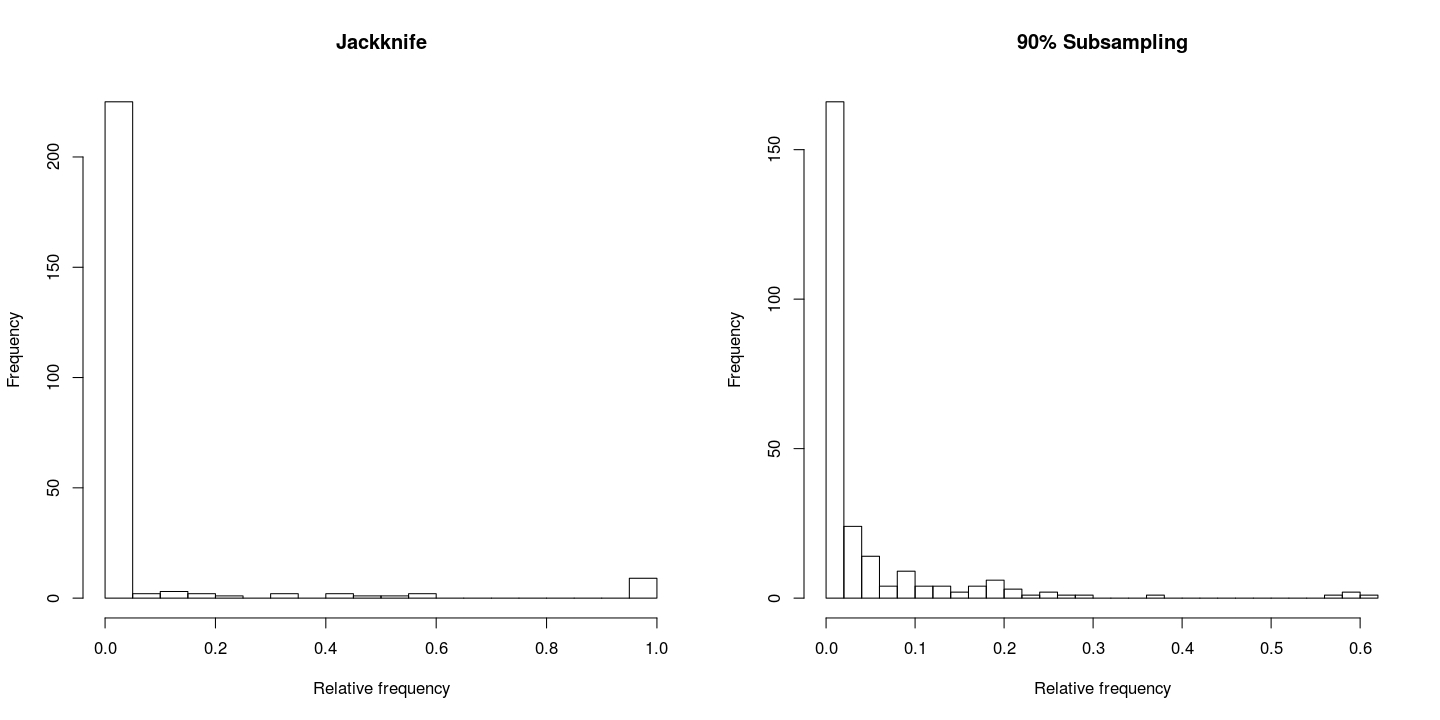

Supplement: Supplementary file 5 [file 813FigureS5.jpg]

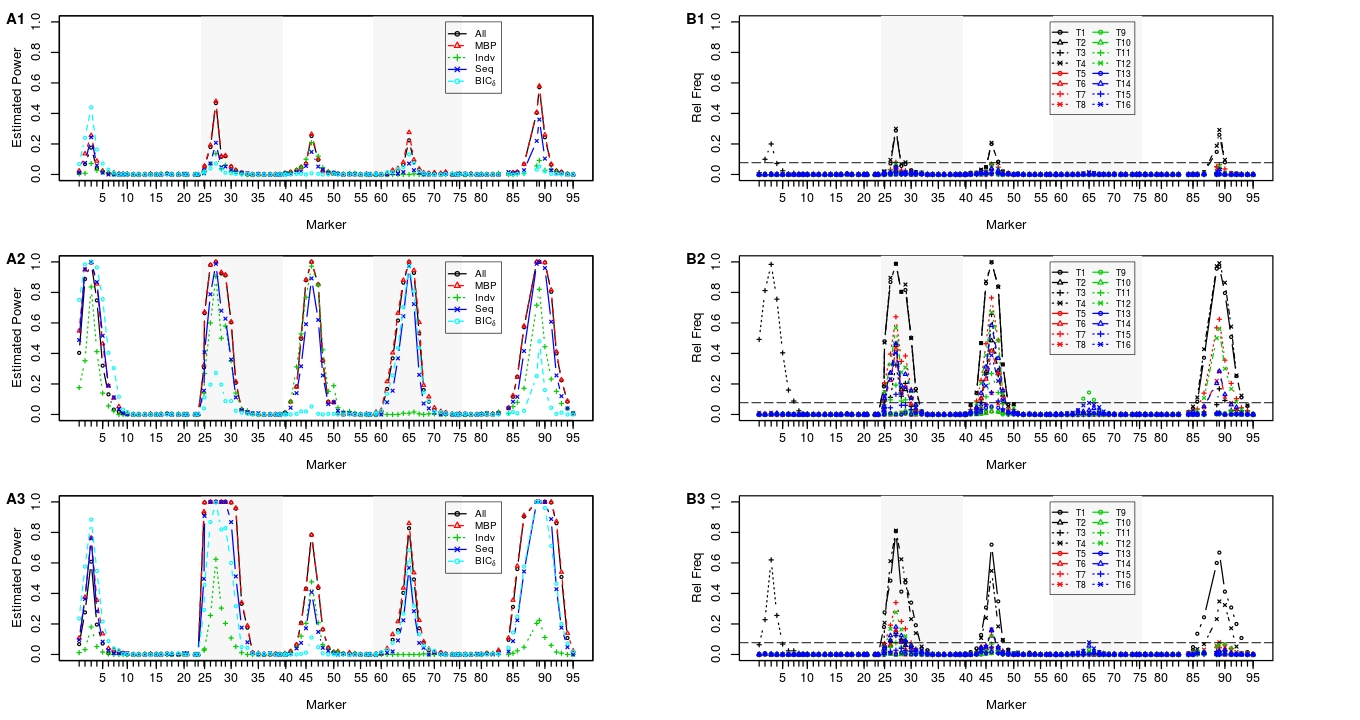

Supplement: Supplementary file 6 [file 813FigureS6.jpg]

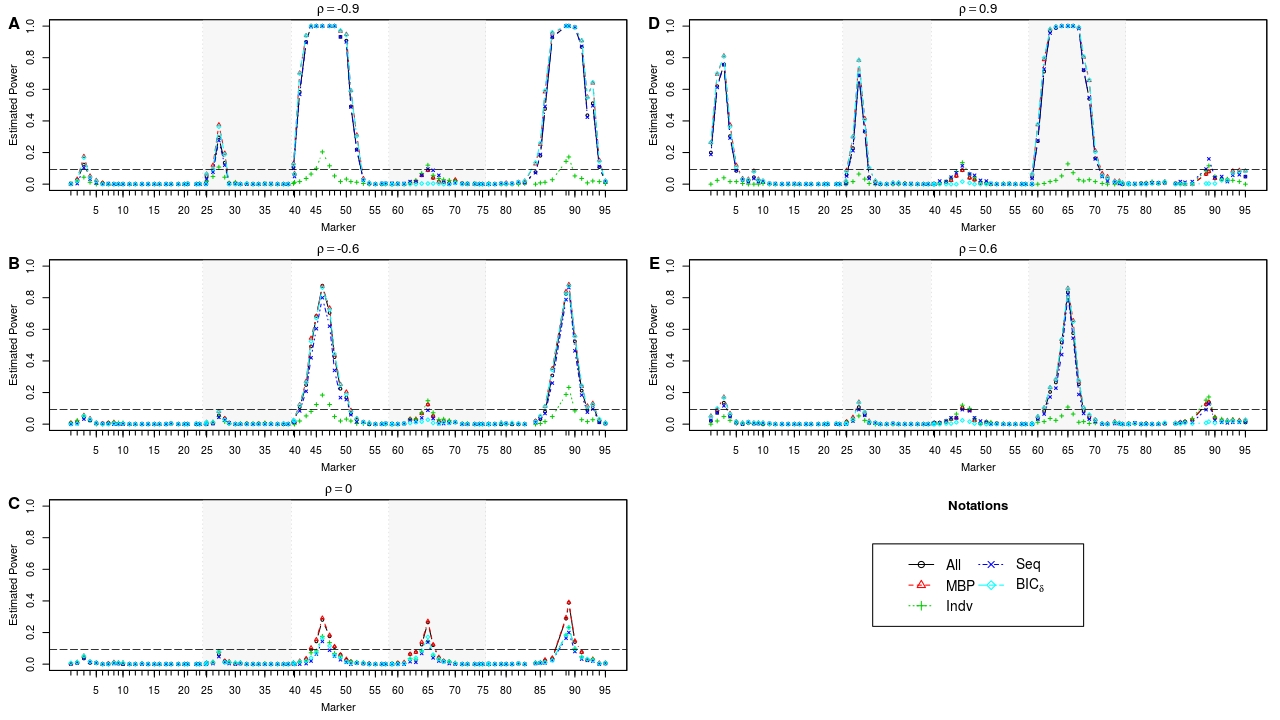

Supplement: Supplementary file 7 [file 813FigureS7.jpg]

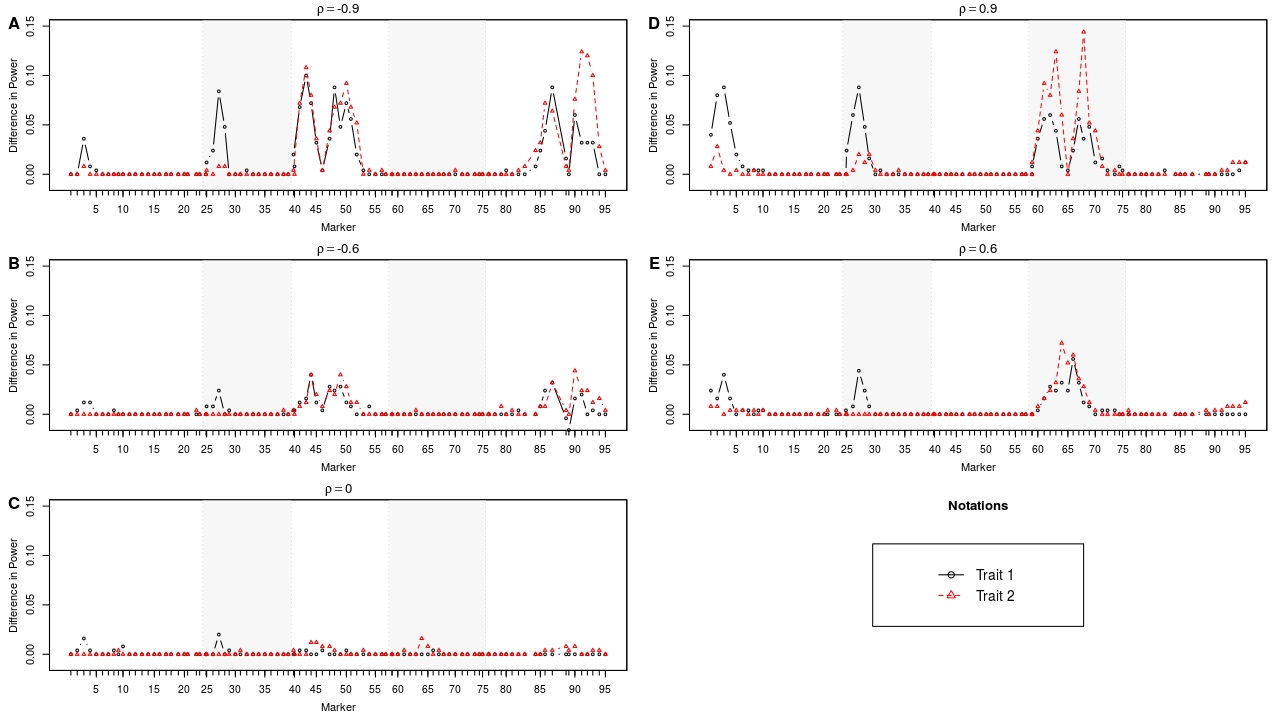

Supplement: Supplementary file 8 [file 813FigureS8.jpg]
