# Supplementary material for: Novel Resampling Improves Statistical Power for Multiple-Trait QTL Mapping
Source: G3 (Bethesda). 2017 Jan 6;7(3):813–22. doi: 10.1534/g3.116.037531 (PMC5345711; doi:10.1534/g3.116.037531)
Supplement: Supplementary file 9 [file 813TableS1.pdf]

.

Table S1 Relative frequency of $\hat{p}$ over 250 simulations that $\hat{p} > 0.25$ by Jackknifing and 90% subsampling.

No. of best QTL Effects	1	2	3	4	5	6	7	8
Jackknife	0.052	0.056	0.040	0.052	0.056	0.048	0.052	0.044
90% Subsampling	0.060	0.060	0.056	0.048	0.052	0.052	0.052	0.060

No. of best QTL Effects	9	10	11	12	13	14	15	16
Jackknife	0.048	0.052	0.056	0.064	0.060	0.064	0.060	0.060
90% Subsampling	0.056	0.048	0.044	0.036	0.032	0.032	0.032	0.032
