# Supplementary material for: Novel Resampling Improves Statistical Power for Multiple-Trait QTL Mapping
Source: G3 (Bethesda). 2017 Jan 6;7(3):813–22. doi: 10.1534/g3.116.037531 (PMC5345711; doi:10.1534/g3.116.037531)
Supplement: Supplementary file 10 [file 813TableS2.pdf]

Table S2 Genome-wide type I error rate (and standard error) estimated from 250 simulations at significance level 0.05 by five methods: All, MBP, Indv, Seq and BIC_δ.

Simulation	All	MBP	Indv	Seq	BIC_δ
1	0.052 (0.014)	0.064 (0.015)	0.032 (0.011)	0.056 (0.015)	0.064 (0.015)
2	0.056 (0.015)	0.056 (0.015)	0.032 (0.011)	0.072 (0.016)	0.064 (0.015)
3	0.044 (0.013)	0.056 (0.015)	0.036 (0.012)	0.072 (0.016)	0.056 (0.015)
