# Supplementary material for: Novel Resampling Improves Statistical Power for Multiple-Trait QTL Mapping
Source: G3 (Bethesda). 2017 Jan 6;7(3):813–22. doi: 10.1534/g3.116.037531 (PMC5345711; doi:10.1534/g3.116.037531)
Supplement: Supplementary file 11 [file 813TableS3.pdf]

Table S3 Simulated QTL effects on two traits at five markers.

Maker	Trait	
	1	2
3	0.3	0
27	-0.3	0
46	0.3	0.3
65	0.3	-0.3
89	-0.3	-0.3
