# Supplementary material for: Novel Resampling Improves Statistical Power for Multiple-Trait QTL Mapping
Source: G3 (Bethesda). 2017 Jan 6;7(3):813–22. doi: 10.1534/g3.116.037531 (PMC5345711; doi:10.1534/g3.116.037531)
Supplement: Supplementary file 12 [file 813TableS4.pdf]

Table S4 Type I error rate (and standard error) estimated from 250 simulations at genome-wide significance level 0.05. Five methods All, MBP, Indv, Seq and BIC_δ were implemented for each of the five correlations between two traits.

Method	Correlation ρ				
	-0.9	-0.6	0	0.6	0.9
All	0.044 (0.013)	0.056 (0.015)	0.056 (0.015)	0.040 (0.012)	0.068 (0.016)
MBP	0.048 (0.014)	0.088 (0.018)	0.080 (0.017)	0.060 (0.015)	0.072 (0.016)
Indv	0.036 (0.012)	0.052 (0.014)	0.052 (0.014)	0.032 (0.011)	0.036 (0.012)
Seq	0.076 (0.017)	0.040 (0.012)	0.020 (0.009)	0.032 (0.011)	0.084 (0.018)
BIC_δ	0.024 (0.010)	0.072 (0.016)	0.064 (0.015)	0.048 (0.014)	0.028 (0.010)
