# Supplementary material for: Novel Resampling Improves Statistical Power for Multiple-Trait QTL Mapping
Source: G3 (Bethesda). 2017 Jan 6;7(3):813–22. doi: 10.1534/g3.116.037531 (PMC5345711; doi:10.1534/g3.116.037531)
Supplement: Supplementary file 13 [file 813TableS5.pdf]

Table S5 (Alternative presentation of Figure S7) Power estimated from 250 simulations at genome-wide significance level 0.05 at marker 3, 27, 46, 65 and 89 where QTL were simulated. Five methods All, MBP, Indv, Seq and BIC_δ were implemented for each of the five correlations between two traits.

Correlation ρ	Method	Marker				
		3	27	46	65	89
-0.9	All	0.124	0.296	1.000	0.088	1.000
	MBP	0.176	0.376	1.000	0.088	1.000
	Indv	0.044	0.108	0.204	0.120	0.172
	Seq	0.104	0.276	1.000	0.096	1.000
	BIC_δ	0.168	0.364	1.000	0.004	1.000
-0.6	All	0.044	0.056	0.876	0.124	0.876
	MBP	0.056	0.080	0.872	0.124	0.884
	Indv	0.052	0.048	0.184	0.148	0.232
	Seq	0.032	0.044	0.800	0.088	0.864
	BIC_δ	0.048	0.080	0.864	0.028	0.876
0.0	All	0.036	0.060	0.280	0.264	0.388
	MBP	0.052	0.080	0.292	0.272	0.392
	Indv	0.044	0.076	0.176	0.144	0.232
	Seq	0.040	0.048	0.144	0.140	0.200
	BIC_δ	0.052	0.076	0.164	0.172	0.236
0.6	All	0.136	0.108	0.104	0.836	0.140
	MBP	0.172	0.140	0.104	0.860	0.140
	Indv	0.048	0.052	0.120	0.108	0.172
	Seq	0.116	0.092	0.092	0.820	0.128
	BIC_δ	0.168	0.136	0.024	0.860	0.016
0.9	All	0.756	0.688	0.088	1.000	0.080
	MBP	0.812	0.784	0.088	1.000	0.080
	Indv	0.040	0.064	0.136	0.128	0.116
	Seq	0.756	0.688	0.116	1.000	0.160
	BIC_δ	0.812	0.792	0.020	1.000	0.004
