# Supplementary material for: Novel Resampling Improves Statistical Power for Multiple-Trait QTL Mapping
Source: G3 (Bethesda). 2017 Jan 6;7(3):813–22. doi: 10.1534/g3.116.037531 (PMC5345711; doi:10.1534/g3.116.037531)
Supplement: Supplementary file 14 [file 813FileS1.pdf]

1 Information about the simulation and the e-trait data

1.1 Variation explained by simulated QTL

As shown in the following plot, non-zero QTL effects explained 0.20$\sim$5.90% of the variation in sixteen simulated traits. Numbers of QTL effects at markers 3, 27, 46, 65 and 89 were 1, 11, 16, 3 and 7 respectively. The largest eleven QTL effects at markers 27 and 46 were the same. However, the power at marker 46 was smaller though there were more QTL effects (see Figures 1 and 2 in the paper). This is an example that more QTL effects do not necessarily yield a better chance for QTL detection.

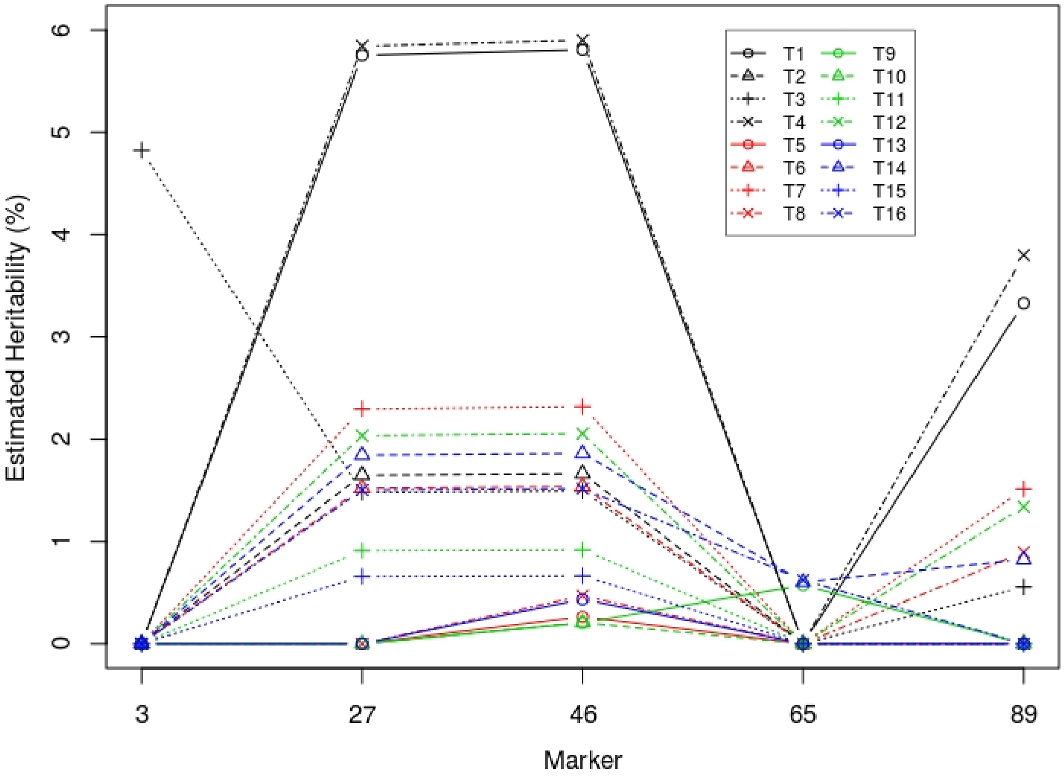

Figure S1 Proportion of the variation in each of the sixteen simulated traits T1, T2, $\cdots$, T16 explained by markers 3, 27, 46, 65 and 89 where QTL were simulated.

1.2 Proportion of 250 simulations that QTL effects were selected

The following figure shows the relative frequencies over 250 simulations that QTL effects were selected for the traits by four methods BMP, Indv, Seq and BIC$_\delta$ at genome-wide significance level 0.05. We can see that all but Indv could not reasonably control the relative frequency at 0.05 when QTL-trait associations did not exist.

Now let's take another look at the problem with under-fitting of QTL effects. Figure S2D shows that marker 27 was more often identified as a QTL for trait 13 than other traits by BIC$_\delta$ thought the marker was not a QTL for this trait (see Figure S1). Marker 89 was apparently more frequently selected for trait 14 than any other trait though the corresponding QTL heritability was only intermediate among the seven QTL effects. These results are not intuitive.

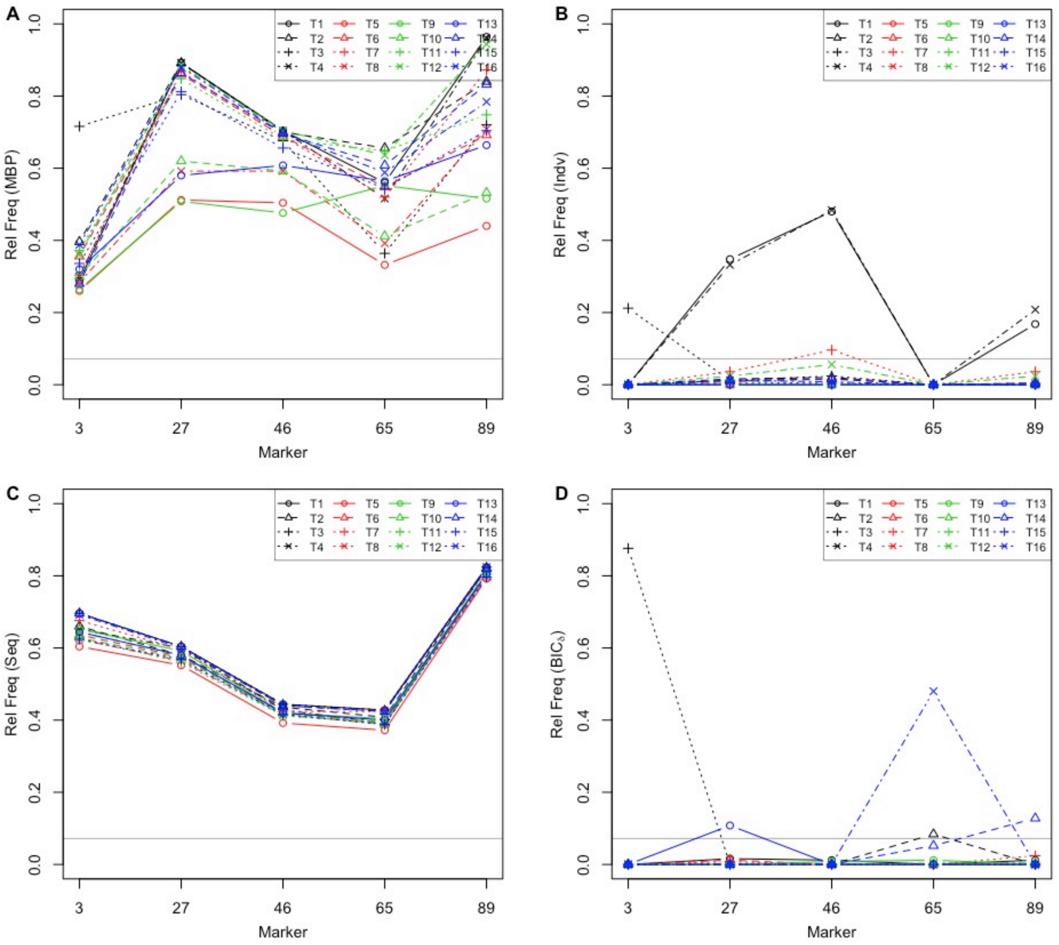

Figure S2 Relative frequency that a QTL effect was selected for the corresponding trait (T1, T2, $\cdots$, or T16) over 250 simulations at genome-wide significance level 0.05, using MBP (A), Indv (B), Seq (C) and BIC$_\delta$ (D). Only the five markers where QTL were simulated are displayed. The horizontal dotted line represents $0.05 + 1.645\sqrt{0.05 \times (1 - 0.05)/250} \approx 0.0727$.

1.3 Proportion of 211 jackknife samples of the e-trait data that each marker was identified as QTL, and the gain by MBP over All

The following plot displays the relative frequencies over 211 jackknife samples of the e-trait data that each marker was identified as QTL by All, MBP, Indv, Seq and BIC$_\delta$. The nominal genome-wide significance level was 0.05. The frequencies in most of the genomic region were either nearly zero or 100%, making comparison among the methods ineffective. Therefore, we scaled the estimated QTL effects in our simulations so that advantage of a method over another in terms of statistical power could be disclosed.

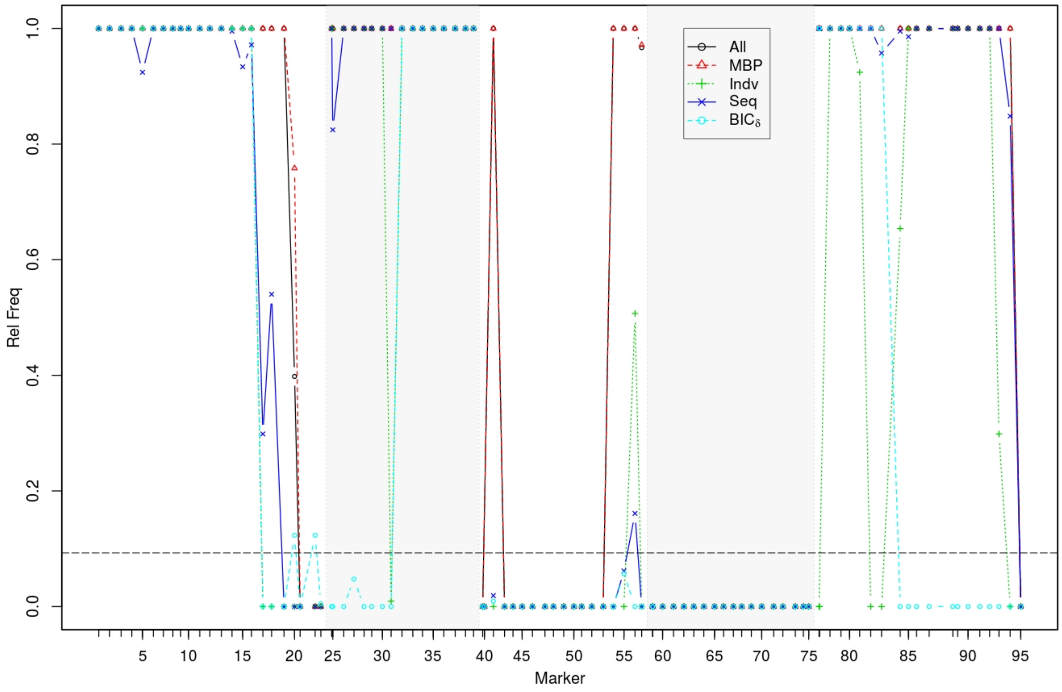

Figure S3 Relative frequency over 211 jackknife samples of the e-trait data that each marker was identified as QTL by All, MBP, Indv, Seq, or BIC$_\delta$.

The difference in the relative frequencies that a scanning locus was identified as QTL for each trait by using MBP+idv and All+idv is shown in the following figure. Obviously, MBP had an advantage over All in many regions.

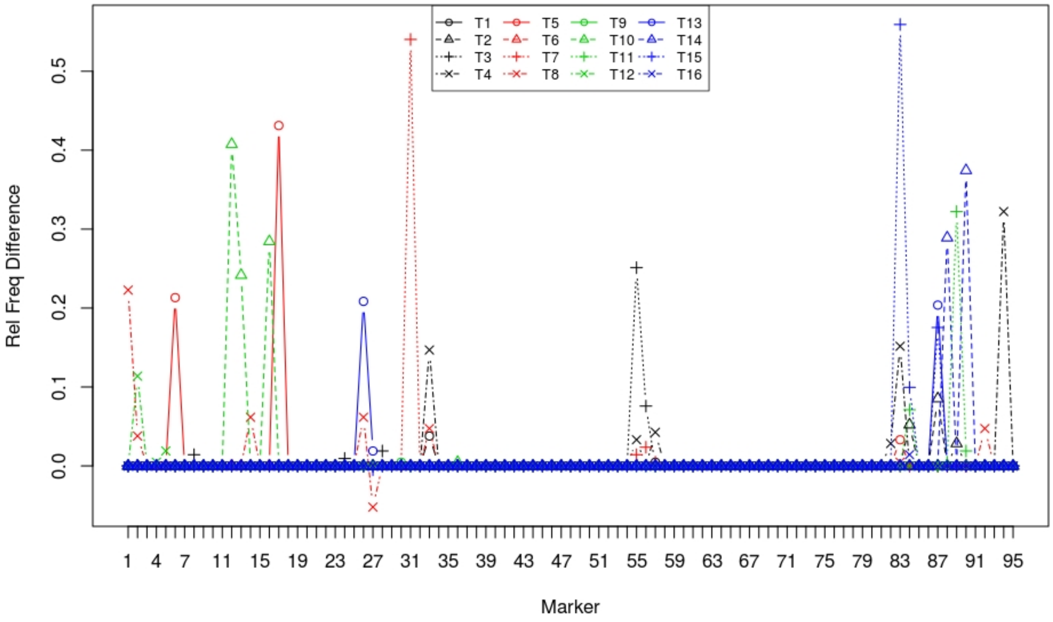

Figure S4 Difference in the relative frequencies over 211 jackknife samples of the e-trait data that a scanning locus was identified as QTL for each of sixteen e-traits T1, T2, · · · , and T16 by using MBP+idv and All+idv.

1.4 The null distribution of relative frequency with respect to resampling

Resampling is expected to reduce uncertainty about QTL identification. However, the distribution of the relative frequency $\hat{p} = \frac{\sum_{1\leq b\leq B} I_{S^{(b)}>\zeta(\alpha)}}{B}$ is not normal, where $S^{(b)} = \max_{1\leq l\leq L} S_l^{(b)}$ with $S_l^{(b)}$ being the test statistic at the l-th of L scanning loci in the b-th of B resamples, and $\zeta(\alpha)$ is the significance threshold at genome-wide significance level α under the assumption that data observations are independent. It does not seem to be known in literature. Therefore, we need to estimate the distribution by simulation. We performed 250 simulations for this purpose. The data was generated in the same way as for studying the type I error rate in the paper. We considered jackknife sampling, 90% subsampling (i.e. randomly draw 90% of the data) and bootstrap. Figure S5 shows histograms of relative frequency over jackknife samples and 90% subsamples when all sixteen QTL effects were tested together. The distribution was extremely skewed to right. The 95-th percentile was 0.244 for jackknife sampling or 0.234 for 90% subsampling. Result was not sensitive to choice of the 95-th percentile in the region around 0.24 so we took 0.25 as a slightly conservative estimate of the 95-th percentile for jackknifing and 90% subsampling. Table S1 shows the relative frequency that $\hat{p} > 0.25$ over 250 simulations when different number of the "best" effects of a putative QTL were tested for QTL existence. The relative frequency for jackknife and 90% subsampling was reasonably close to 0.05, meaning that 0.25 was a reasonably estimate of 0.05 significance threshold. This threshold does not seem to depend on whether the test is performed at single scanning loci or genome-wide or on the number of the best QTL effects. Instead, what matters seems to be the fact that $I_{S^{(b)}>\zeta(\alpha)}$ is a binomial variable with $P(I_{S^{(b)}>\zeta(\alpha)} = 1) = P(S^{(b)} > \zeta(\alpha)) = \alpha$. Of course, this conjecture is subject to theoretical proof. Bootstrap yielded minimum relative frequency 0.772 and minimum 95-th percentile 0.986. The distribution of relative frequency was seriously skewed to left so that the 95-th percentile could not be appropriately estimated from a limited number of simulations. At genome-wide significance level $\alpha = 0.05$, $\zeta(\alpha)$ resulted in a mean relative frequency (i.e. the bootstrap relative frequency averaged over 250 simulations) at single scanning loci ranging from 0.187 to 0.232 when all sixteen effects of a putative QTL were tested together.

Table S1 Relative frequency of $\hat{p}$ over 250 simulations that $\hat{p} > 0.25$ by Jackknifing and 90% subsampling.

No. of best QTL Effects	1	2	3	4	5	6	7	8
Jackknife	0.052	0.056	0.040	0.052	0.056	0.048	0.052	0.044
90% Subsampling	0.060	0.060	0.056	0.048	0.052	0.052	0.052	0.060

No. of best QTL Effects	9	10	11	12	13	14	15	16
Jackknife	0.048	0.052	0.056	0.064	0.060	0.064	0.060	0.060
90% Subsampling	0.056	0.048	0.044	0.036	0.032	0.032	0.032	0.032

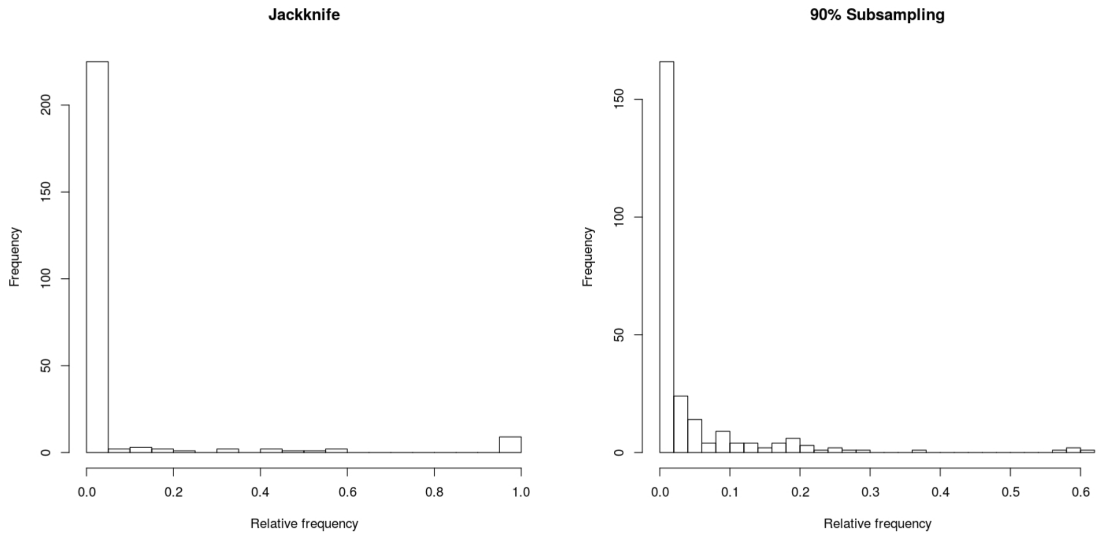

Figure S5 Histograms of relative frequency obtained from 211 jackknife samples (left panel) and 250 90% subsamples (right panel) that QTL was identified at genome-wide significance level 0.05 in 250 simulations when there was no QTL.

2 Consolidating simulation studies

Bias toward our method MBP is something we took great care of. To avoid it, we chose to use the sampling variance-covariance and estimated QTL effects. We simply scaled the estimated QTL effects and truncated some effects. The scaling was to make some adjustment so that the gain could be disclosed. The truncating was to make different scenarios that represent some common situations with different potentials for gain in power. Other than these, there was nothing in favor of MBP. Rather, the small QTL heritabilities after scaling imposed difficulties as it is generally hard to definitely measure the contribution of a small QTL effect. Also note that after scaling and truncating, the QTL heritatilities ranged 0.20-5.9%, covering haritabilities most commonly estimated in practice.

Now we present more simulation studies to further support our proposed method in terms of both type I error rates and statistical power.

2.1 Sixteen traits with variance-covariance being the sample variance-covariance of the e-traits

We did three additional sets of simulations that again used the sample variance-covariance of the sixteen e-traits to check if the type I error rate was controlled at a nominal significance level. The simulation settings were the same as the corresponding settings in the paper. Results are shown in Table S2 and look reasonable.

Table S2 Genome-wide type I error rate (and standard error) estimated from 250 simulations at significance level 0.05 by five methods: All, MBP, Indv, Seq and BIC$_\delta$.

Simulation	All	MBP	Indv	Seq	BIC$_\delta$
1	0.052 (0.014)	0.064 (0.015)	0.032 (0.011)	0.056 (0.015)	0.064 (0.015)
2	0.056 (0.015)	0.056 (0.015)	0.032 (0.011)	0.072 (0.016)	0.064 (0.015)
3	0.044 (0.013)	0.056 (0.015)	0.036 (0.012)	0.072 (0.016)	0.056 (0.015)

Yet, we did another three additional sets of simulations that used the sample variance-covariance of the sixteen e-traits to validate the conclusions about the performances of the methods in terms of statistical power in the paper. We were especially interested in comparing All and MBP and how well MBP was able to identify trait-specific QTL at controlled type I error rates. The three sets of simulations were set respectively as follows.

1) For $k = 1, 2, \cdots, 16$, we defined $\gamma_{lk} = \begin{cases} \hat{\beta}_{lk}/4 & \text{if } |\hat{\beta}_{lk}|/\sqrt{\hat{\sigma}_{kk}} \geq 0.45 \\ 0 & \text{if } |\hat{\beta}_{lk}|/\sqrt{\hat{\sigma}_{kk}} < 0.45 \end{cases}$, $l \in \{3, 27, 65, 89\}$, and $\gamma_{46,k} = \hat{\beta}_{27,k}/4$. All other setting were the same as in the paper.

2) For $k = 1, 2, \cdots, 16$, we defined $\gamma_{lk} = \begin{cases} \hat{\beta}_{lk}/2 & \text{if } |\hat{\beta}_{lk}|/\sqrt{\hat{\sigma}_{kk}} \geq 0.45 \\ 0 & \text{if } |\hat{\beta}_{lk}|/\sqrt{\hat{\sigma}_{kk}} < 0.45 \end{cases}$, $l \in \{3, 27, 65, 89\}$, and $\gamma_{46,k} = \hat{\beta}_{27,k}/2$. All other setting were the same as in the paper.

3) For $k = 1, 2, \cdots, 16$, we defined $\gamma_{lk} = \begin{cases} \delta_k\hat{\beta}_{lk}/3 & \text{if } |\hat{\beta}_{lk}|/\sqrt{\hat{\sigma}_{kk}} \geq 0.45 \\ 0 & \text{if } |\hat{\beta}_{lk}|/\sqrt{\hat{\sigma}_{kk}} < 0.45 \end{cases}$, $l \in \{3, 27, 65, 89\}$, and $\gamma_{46,k} = \delta_k\hat{\beta}_{27,k}/3$ where δ_k was randomly pre-defined as 1 or -1

Results of 250 simulations are shown in Figure S6 with the panel numbers 1-3 corresponding to the above settings 1-3. Basically, MBP had more power than All across all the markers where QTL were simulated (panels A1-3 in Figure S6). The gain in power was up to 16%, depending on QTL effects (or more accurately heritability) and the number of QTL effects. As desired, the type I error rate for identification of QTL for a specific trait was reasonably controlled at the nominal significance level, disregarding the number of QTL effects, even if MBP had a power of 100% at the locus (panels B1-3 in Figure S6). This again means that the proposed method MBP+idv is powerful for identifying trait-specific QTL in multiple-trait analysis at controlled type I error rates.

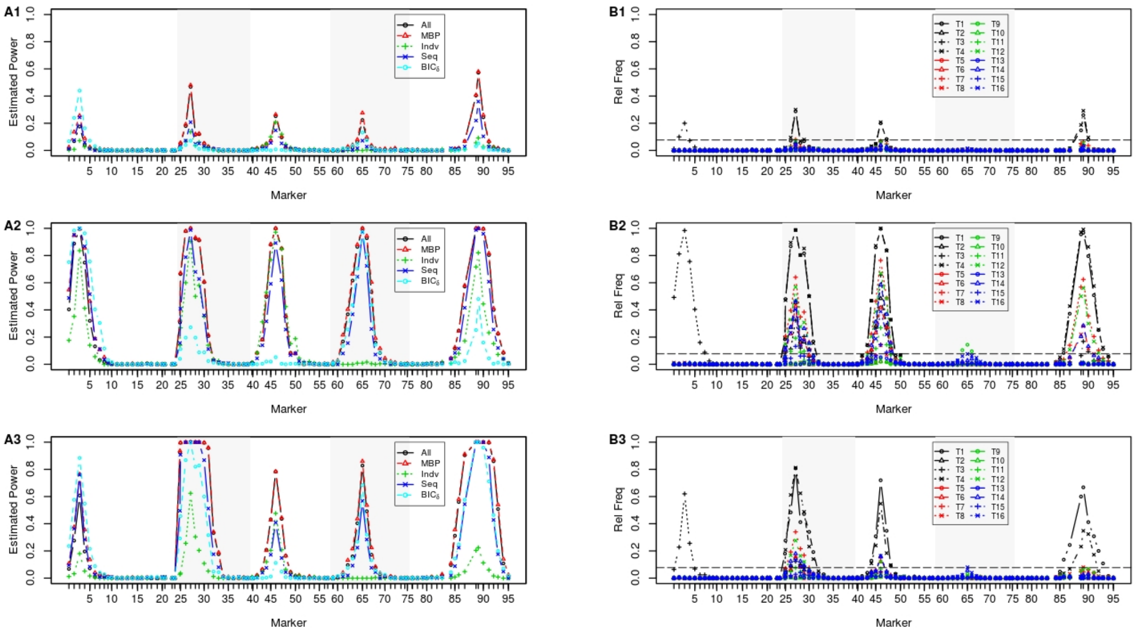

Figure S6 Proportion of 250 simulations that a marker was identified as QTL by five methods: All, MBP, Indv, Seq and BIC$_\delta$ (panels A1-3) or was identified as QTL for each trait by MBP+idv (panels B1-3).

2.2 Two traits with various correlations

Whether our proposed method MBP leads to a gain in power and how much the gain is depend on many factors such as the number of traits and the number of QTL effects, the heritability of QTL, the correlation structure, etc. Figure 1 shows some scenarios representing a few common situations and the potential for power gain. Of course, our method can not attain the full potential. Next we simulate two traits with various correlations and show that there is likely a substantial gain in power even in two-trait case.

We used the genotypes in the e-trait data and simulated two traits whose residuals identically independently followed the standard normal distribution $N(0,1)$. When we studied statistical power, we placed five QTL at markers 3, 27, 46, 65 and 89 with effects shown in Table S3. The correlation between the traits was -0.9, -0.6, 0, 0.6 or 0.9. Other cases may be derived from these settings. For instance, if a QTL has an effect 0.3 on trait 1 and an effect 0.1 on trait 2, then the power should be somewhere between those at markers 3 and 46. Both the type I error rate and statistical power were estimated from 250 simulations by five methods All, MBP, Indv, Seq, and BIC$_\delta$ that are defined in the paper. Overall, the type I error rate was reasonably controlled at the nominal genome-wide significance level 0.05 (Table S4), and the relative performances of the five methods in terms of statistical power were in line with what we observed in the paper (Figure S7 or Table S5). Of course, the advantage or disadvantage of a method might not be as prominent as when the number of traits was relatively large. Nonetheless, the simulations showed that MBP could be more powerful than All (by up to 12.8%, or 9.6% at markers where QTL were simulated; see Table S5) even when there were two traits. The gain in power for QTL-trait associations was more prominent (see Figure S8). It was not necessarily at markers where QTL were simulated but at nearby linked markers; however, the configurations regarding QTL effects and correlation structures likely occur in applications.

Table S3 Simulated QTL effects on two traits at five markers.

Maker	Trait	
	1	2
3	0.3	0
27	-0.3	0
46	0.3	0.3
65	0.3	-0.3
89	-0.3	-0.3

Table S4 Type I error rate (and standard error) estimated from 250 simulations at genome-wide significance level 0.05. Five methods All, MBP, Indv, Seq and BIC$_\delta$ were implemented for each of the five correlations between two traits.

Method	Correlation ρ				
	-0.9	-0.6	0	0.6	0.9
All	0.044 (0.013)	0.056 (0.015)	0.056 (0.015)	0.040 (0.012)	0.068 (0.016)
MBP	0.048 (0.014)	0.088 (0.018)	0.080 (0.017)	0.060 (0.015)	0.072 (0.016)
Indv	0.036 (0.012)	0.052 (0.014)	0.052 (0.014)	0.032 (0.011)	0.036 (0.012)
Seq	0.076 (0.017)	0.040 (0.012)	0.020 (0.009)	0.032 (0.011)	0.084 (0.018)
BIC$_\delta$	0.024 (0.010)	0.072 (0.016)	0.064 (0.015)	0.048 (0.014)	0.028 (0.010)

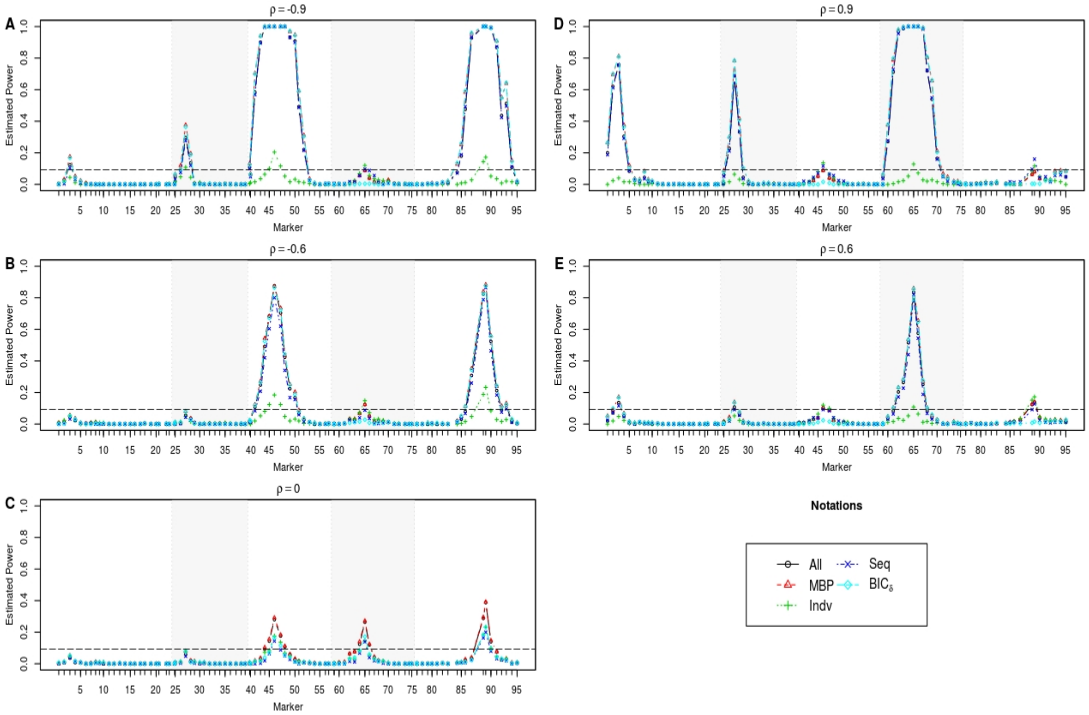

Figure S7 Proportion over 250 simulations that a marker was identified as QTL at genome-wide significance level 0.05 by five methods: All, MBP, Indv, Seq and BIC$_\delta$.

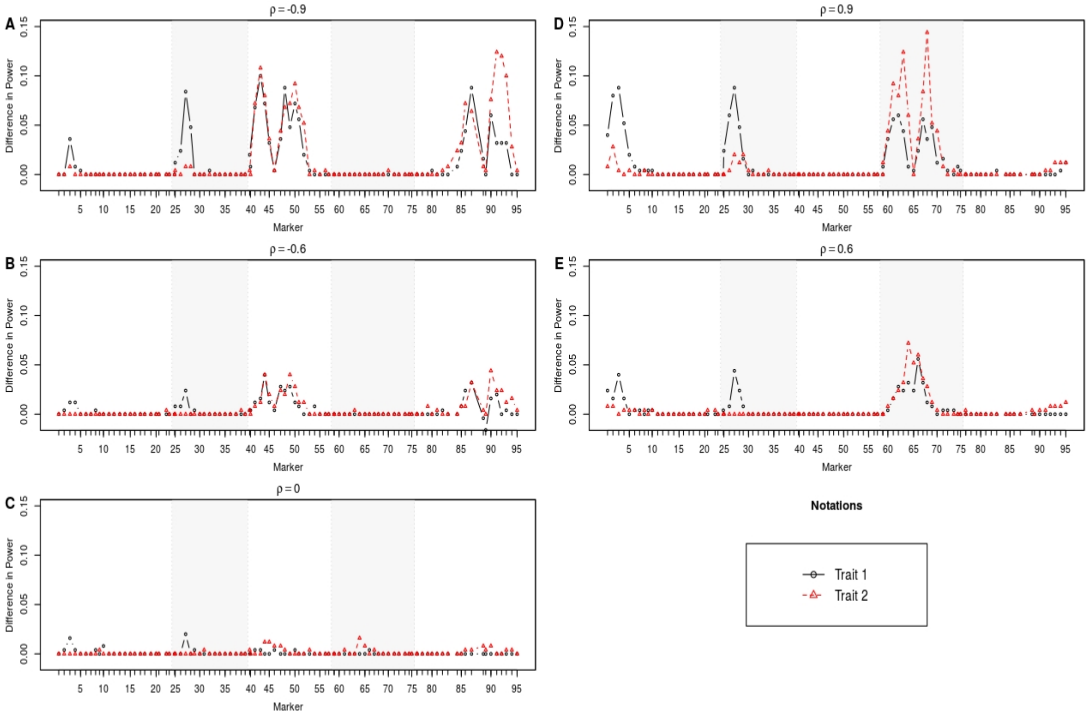

Figure S8 Difference in the power for QTL-trait associations by using MBP+idv and All+idv.

Table S5 (Alternative presentation of Figure S7) Power estimated from 250 simulations at genome-wide significance level 0.05 at marker 3, 27, 46, 65 and 89 where QTL were simulated. Five methods All, MBP, Indv, Seq and BIC$_\delta$ were implemented for each of the five correlations between two traits.

Correlation ρ	Method	Marker				
		3	27	46	65	89
-0.9	All	0.124	0.296	1.000	0.088	1.000
	MBP	0.176	0.376	1.000	0.088	1.000
	Indv	0.044	0.108	0.204	0.120	0.172
	Seq	0.104	0.276	1.000	0.096	1.000
	BIC$_\delta$	0.168	0.364	1.000	0.004	1.000
-0.6	All	0.044	0.056	0.876	0.124	0.876
	MBP	0.056	0.080	0.872	0.124	0.884
	Indv	0.052	0.048	0.184	0.148	0.232
	Seq	0.032	0.044	0.800	0.088	0.864
	BIC$_\delta$	0.048	0.080	0.864	0.028	0.876
0.0	All	0.036	0.060	0.280	0.264	0.388
	MBP	0.052	0.080	0.292	0.272	0.392
	Indv	0.044	0.076	0.176	0.144	0.232
	Seq	0.040	0.048	0.144	0.140	0.200
	BIC$_\delta$	0.052	0.076	0.164	0.172	0.236
0.6	All	0.136	0.108	0.104	0.836	0.140
	MBP	0.172	0.140	0.104	0.860	0.140
	Indv	0.048	0.052	0.120	0.108	0.172
	Seq	0.116	0.092	0.092	0.820	0.128
	BIC$_\delta$	0.168	0.136	0.024	0.860	0.016
0.9	All	0.756	0.688	0.088	1.000	0.080
	MBP	0.812	0.784	0.088	1.000	0.080
	Indv	0.040	0.064	0.136	0.128	0.116
	Seq	0.756	0.688	0.116	1.000	0.160
	BIC$_\delta$	0.812	0.792	0.020	1.000	0.004
